# Mimicking H3 Substrate Arginine in the Design of G9a Lysine Methyltransferase Inhibitors for Cancer Therapy: A Computational Study for Structure-Based Drug Design

**DOI:** 10.1021/acsomega.0c04710

**Published:** 2021-02-22

**Authors:** M. Ramya Chandar Charles, Mu-Chun Li, Hsing-Pang Hsieh, Mohane Selvaraj Coumar

**Affiliations:** † Centre for Bioinformatics, School of Life Sciences, Pondicherry University, Kalapet, Puducherry 605014, India; ‡ Institute of Biotechnology and Pharmaceutical Research, National Health Research Institutes, 35 Keyan Road, Zhunan, Miaoli County, Taiwan 350, ROC; § Department of Chemistry, National Tsing Hua University, No. 101, Section 2, Kuang-Fu Road, Hsinchu 300, Taiwan; ∥ Biomedical Translation Research Center, Academia Sinica, Taipei 115, Taiwan

## Abstract

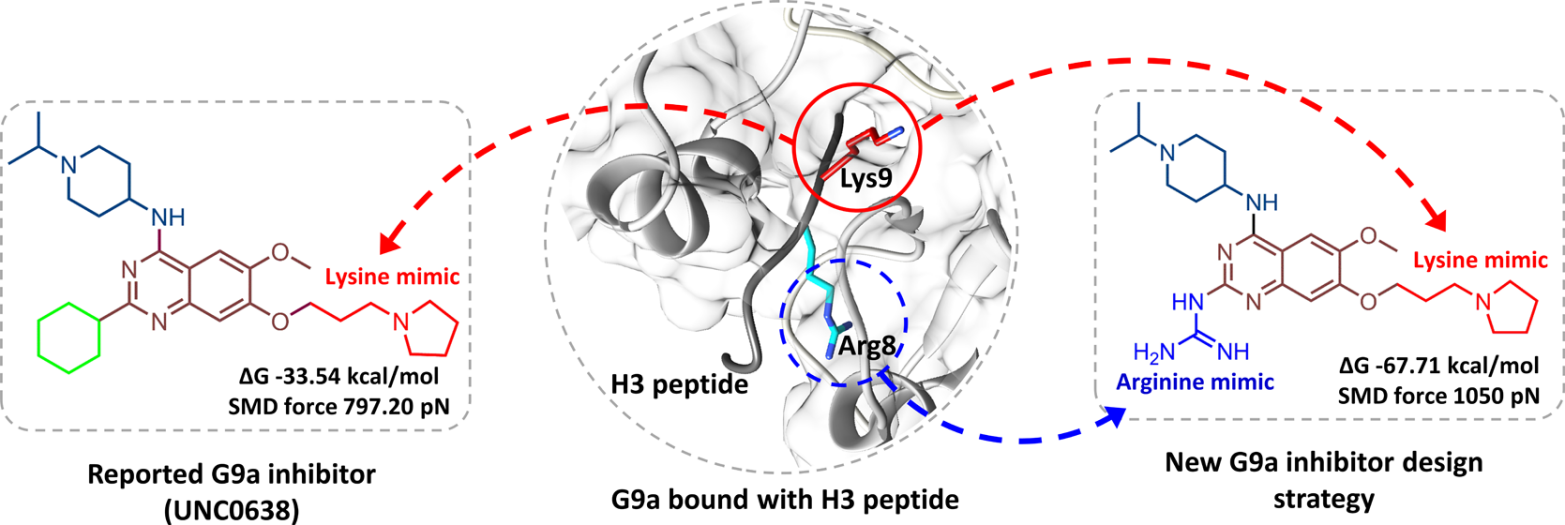

G9a protein methyltransferase is a potential epigenetic drug target in different cancers and other disease conditions overexpressing the enzyme. G9a is responsible for the H3K9 dimethylation mark, which epigenetically regulates gene expression. Arg8 and Lys9 of the H3 substrate peptide are the two crucial residues for substrate-specific recognition and methylation. Several substrate competitive inhibitors are reported for the potent inhibition of G9a by incorporating lysine mimic groups in the inhibitor design. In this study, we explored the concept of arginine mimic strategy. The hydrophobic segment of the reported inhibitors BIX-01294 and UNC0638 was replaced by a guanidine moiety (side-chain moiety of arginine). The newly substituted guanidine moieties of the inhibitors were positioned similar to the Arg8 of the substrate peptide in molecular docking. Additionally, improved reactivity of the guanidine-substituted inhibitors was observed in density functional theory studies. Molecular dynamics, molecular mechanics Poisson–Boltzmann surface area binding free energy, linear interaction energy, and potential mean force calculated from steered molecular dynamics simulations of the newly designed analogues show enhanced conformational stability and improved H-bond potential and binding affinity toward the target G9a. Moreover, the presence of both lysine and arginine mimics together shows a drastic increase in the binding affinity of the inhibitor towards G9a. Hence, we propose incorporating a guanidine group to imitate the substrate arginine’s side chain in the inhibitor design to improve the potency of G9a inhibitors.

## 1 Introduction

**Figure 1:**
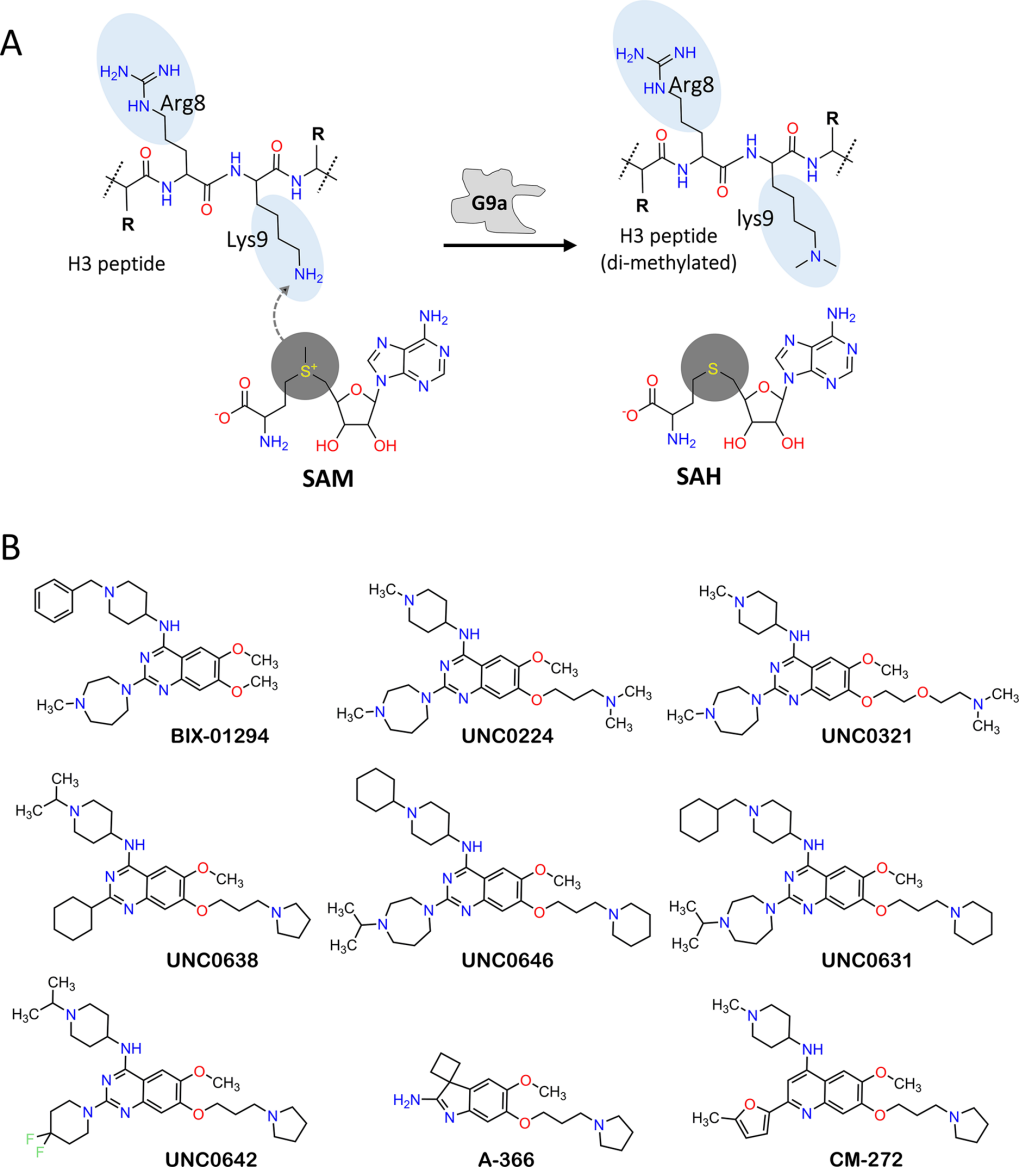
(A) Scheme depicting the catalytic reaction performed by G9a lysine methyltransferase: substrate H3K9 peptide is dimethylated by G9a in which the methyl group donor SAM is converted to SAH. (B) Substrate-competitive G9a inhibitors. The majority of the reported inhibitors possess a lysine mimicking group.

G9a protein methyltransferase, also known as EHMT2 (euchromatin histone methyltransferase 2) and KMT1C (lysine methyltransferase 1C), is primarily responsible for the dimethylation of H3K9 and many other nonhistone substrates; the cofactor SAM (S-adenosyl methionine) acts as the methyl group donor (Figure 1A).^1,2^ G9a regulates several biological processes such as DNA methylation,^2^ chromatin remodeling, transcriptional regulation,^2^ proliferation, differentiation, apoptosis, tumor cell movement,^3−6^ and HIV latency.^7,8^ Besides, G9a is also involved in cellular reprogramming,^9^ embryonic development,^10^ prevention of cardiac hypertrophy,^11^ occurrences of Alzheimer’s disease,^12^ retinoic acid signaling,^13^ repairing of damaged DNA,^14^ intellectual and cognitive disturbances.^15^ Owing to G9a′s diverse role in the maintenance of physiological state and its involvement in the various pathophysiological states, it has become an important drug target. Several substrate-competitive inhibitors of G9a are reported (Figure 1B), particularly for cancer treatment.

**Figure 2:**
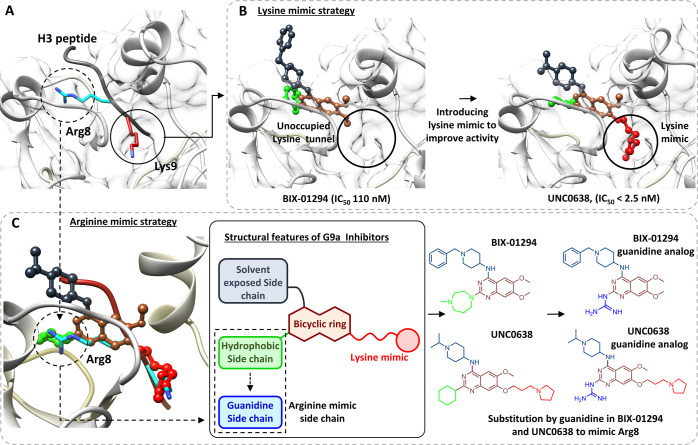
SBDD strategy for developing G9a inhibitors based on G9a–H3 substrate peptide interactions. (A) H3 substrate peptide-bound G9a. Only residues Lys9 and Arg8 are shown for clarity. (B) Lysine mimic strategy: introduction of lysine mimic side chain similar to substrate peptide Lys9 on quinazoline ring improves the potency toward G9a in the inhibitor UNC0638. (C) Arginine mimic strategy: introduction of the guanidine group to mimic the substrate peptide Arg8 to improve the activity of BIX-01294 and UNC0638.

Since the exploration of protein lysine methyltransferases during the early 2000s, chaetocin and BIX-01294 were reported as inhibitors of SU(VAR)3-9 and G9a.^16^ Until the identification of chaetocin, analogues of the methyl donor SAM (cofactor) such as S-adenosyl homocysteine (SAH) and methylthio adenosine were used as inhibitors of methyltransferases.^17^ BIX-01294 is the first selective substrate competitive inhibitor of G9a/GLP discovered through high throughput screening.^18^ The crystal structure of GLP-bound BIX-01294 reveals that the target’s lysine tunnel was unoccupied by the inhibitor BIX-01294. The G9a lysine tunnel binds and accommodates the substrate peptide H3K9 lysine group for methylation reaction. Noticing this, two different groups independently reported the concept of introducing side chains in BIX-01294 to mimic the lysine residue so that the lysine mimic side chain would occupy the lysine tunnel of G9a. UNC0224 and E72 are the two G9a inhibitors initially reported possessing a lysine mimic in their quinazoline core.^19−21^ UNC0224 is the structural derivative of BIX-01294 designed by introducing the dimethylaminopropoxy group as the lysine mimic at the 7-position of the quinazoline ring, which binds to G9a with the lysine tunnel occupied.^21^ Moreover, superimposition of the G9a structures bound with the H3 substrate peptide and UNC0224 shows that the lysine mimic introduced in the inhibitor is positioned similar to the lysine residue (Lys9) of the H3 substrate peptide.^19^ UNC0224 showed more than twofold improved G9a inhibitory activity over BIX-01294. Later, several G9a inhibitors (Figure 1B), UNC0321, UNC0631, UNC0638, UNC0642, UNC0646, A-366, and CM-272, were designed to possess lysine mimic side chains to improve the selectivity and G9a potency.^20,22−26^ X-ray crystallography showed that the lysine mimic side chain of UNC0638 (Figure 2B) and A-366 also occupied the lysine tunnel of G9a, similar to that of UNC0224. CM-272 possesses a quinoline ring instead of a quinazoline ring and is reported to be a dual inhibitor of G9a and DMNT1.^26^ Although several compounds are reported as G9a inhibitors, none of these G9a inhibitors could reach clinical stage due to several reasons, including poor membrane permeability and lack of sufficient potency *in vivo*.^27^ Here, in this study, we attempt to design new chemotypes using substrate structure-based approaches for better G9a modulation that would have potential for development as drug molecules. Different anticancer drug targets require different levels of affinity range to show effective and clinically relevant therapeutic potential.^28,29^ Structure-based inhibitor design usually deals with the conceptualization of molecules that have a highly complementary shape to their enzyme or protein-receptor active site. Affinity is the first step prior to having appropriate ADMET properties. Most perfect complementary inhibitor geometries and noncovalent interactions in the binding site ensure the improved selectivity and affinity of the inhibitors.^30^

G9a recognizes heptapeptide (7–11th residues) residues of H3. Earlier, biochemical studies showed that the mutation of Arg8 of the H3 substrate peptide to any residue abolishes the methylation activity by G9a.^31^ Our computational investigations were performed to understand the architecture of G9a active site also reveal the importance of Arg8 in the substrate peptide binding to G9a.^32^ Also, the superimposition of the H3 substrate peptide over G9a inhibitors (BIX-01294 and UNC0638) bound to G9a showed that Arg8’s guanidine side chain binds in the region where the hydrophobic chain of G9a inhibitors bind (Figure 2C). Based on these findings, we substitute the hydrophobic side chain of the G9a inhibitors with a guanidine group to mimic the Arg8 of the H3 substrate peptide. The guanidine side chain in the inhibitor could improve the inhibitors’ binding to G9a, thereby increasing the potency. Based on this arginine mimic strategy, the guanidine group was substituted on BIX-01294 and UNC0638 (Figure 2C), and the designed compounds were evaluated *in silico* using docking and molecular dynamics simulations. The computational studies show that the newly designed compounds with a guanidine group, BIX-01294 guanidine analogue, and UNC0638 guanidine analogue have better interaction, conformational stability and binding energy with G9a than the BIX-01294 and UNC0638, respectively.

## 2 Results and Discussion

Our previous computational investigations of G9a–substrate peptide complexes revealed the essentiality of the Arg8 residue of the H3 substrate peptide for binding to G9a, which was well correlated with the reported biochemical studies.^31^ Hence, we hypothesized and designed BIX-01294 and UNC0638 guanidine analogues to mimic the guanidine group of Arg8. The guanidine mimic strategy was devised similar to the earlier reported lysine mimic strategy to improve the binding with the G9a target protein.^33^

**Figure 3:**
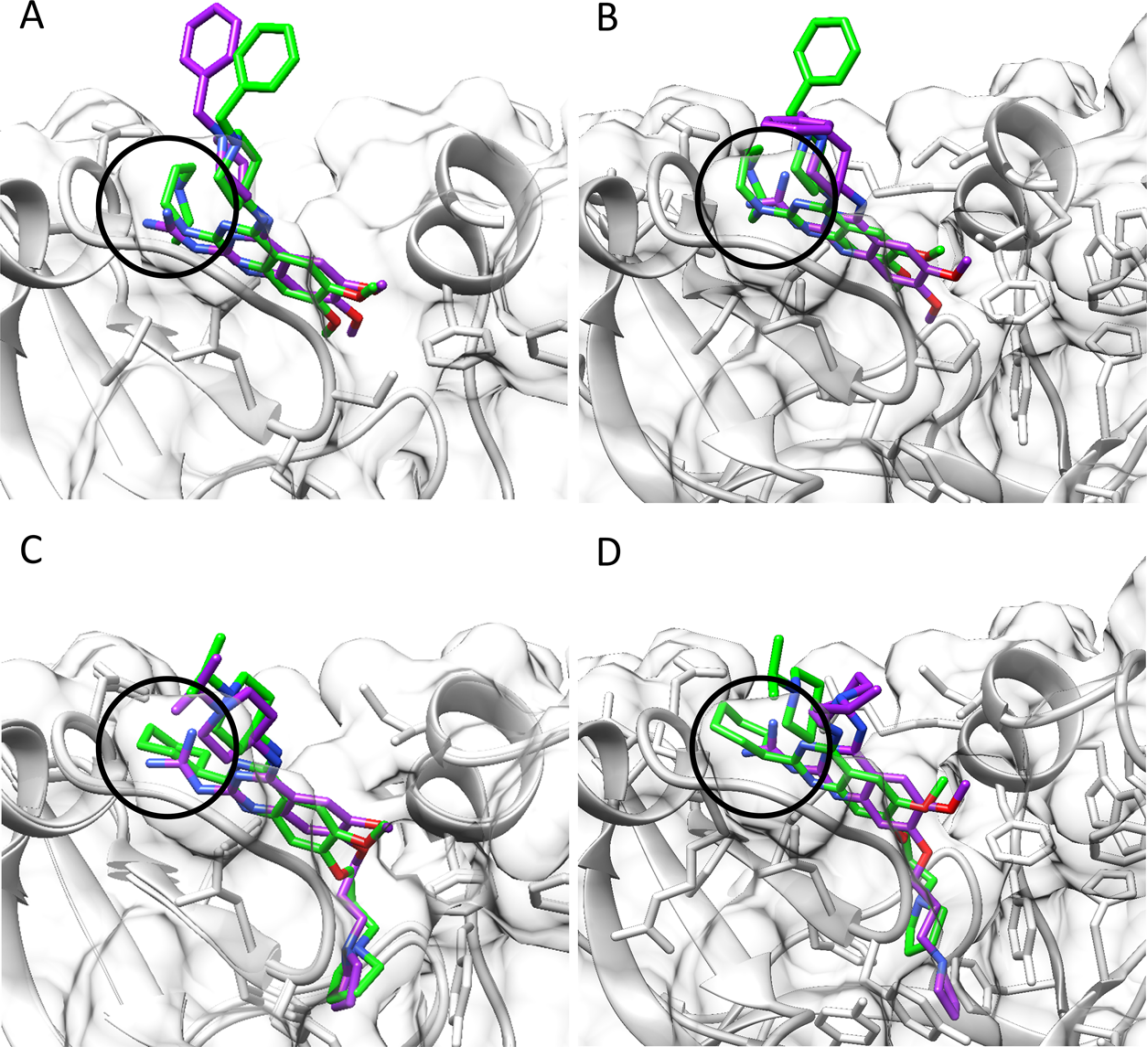
Docked orientation of newly designed guanidine analogues in G9a (PDB ID: 3RJW). Superimposition of glide XP docked orientation of the (A) BIX-01294 (green) and BIX-01294 guanidine analogue (magenta) and (C) UNC0638 (green) and UNC0638 guanidine analogue (magenta). Superimposition of inducted fit docked orientation of (B) BIX-01294 (green) and BIX-01294 guanidine analogue (magenta) and (D) UNC0638 (green) and UNC0638 guanidine analogue. The black circle highlights the guanidine group’s position in the newly designed analogues with respect to the hydrophobic group in the original ligands (BIX-01294 and UNC0638). Docked complex PDB files are given as the Supporting Information, PDB-S1–S4.

To predict the newly designed guanidine analogues’ binding orientation, compounds were docked along with the original inhibitors (BIX-01294 and UNC0638) to the G9a crystal structure (PDB ID: 3RJW). The guanidine analogues’ binding conformations were quite similar to the original inhibitors in the G9a active site. Moreover, the newly introduced guanidine groups were found to occupy the place where the hydrophobic groups were present in the original ligands (Figure 3). Also, the number of H-bonds formed between the inhibitor and G9a were increased or maintained in the case of guanidine analogues. The guanidine analogues formed new H-bonds with Asp1078 through the guanidine group, which was not present in the original ligands. However, the glide docking score for the guanidine analogues was lower than that for the original inhibitors (Table 1). Hence, induced fit docking of the compounds was performed, which showed that the newly designed guanidine analogues were able to bind deeper in the G9a active site when the active site residues were made flexible and also exhibited better docking score than the original inhibitors (Table 1). The binding orientations of the docked guanidine analogues are similar to their corresponding known inhibitors (Figure 3).

**Table 1:** Computational Profile of Newly Designed Guanidine Analogues, in Comparison with Known G9a Inhibitors

compound	docking score^a^ (kcal/mol)	docking score^b^ (kcal/mol)	HOMO LUMO energy gap Δ*E*^c^ (eV)	binding energy^d^ (kcal/mol)	binding energy^e^ (kcal/mol)	rupture force^f^ (pN)
BIX-01294	–8.674	–9.606	0.14496	–33.44 ± 6.32	–57.4798	815 ± 116.600
BIX-01294 guanidine analogue	–7.427	–10.256	0.138966	–52.57 ± 10.06	–66.4265	934 ± 171.996
UNC0638	–13.781	–14.406	0.148	–33.54 ± 5.27	–51.3628	797.20 ± 56.080
UNC0638 guanidine analogue	–12.070	–14.839	0.140531	–67.71 ± 10.10	–117.2585	1050 ± 169.134

a Glide XP docking.

b Induced fit glide docking.

c DFT calculation.

d MMPBSA energy calculation.

e Linear interaction energy.

f Steered molecular dynamics.

For the inhibitors and guanidine analogues, structural and electronic properties were calculated using density functional theory (DFT).^34^ Molecular orbitals provide information about the small molecules’ electronic distribution, which can be correlated with the molecules’ reactivity and stability. The higher- and lower-electron density regions’ highest occupied and lowest unoccupied molecular orbitals (HOMO and LUMO) on the inhibitors and guanidine analogues were computed to imply the compounds’ reactive potential.^34^ The smaller energy gap between these HOMO and LUMO orbitals often directly correlates with the reactivity.^35^ The HOMO–LUMO energy gap of BIX-01294 and UNC0638 were 0.145 and 0.148 eV, respectively, and this orbital energy gap was further reduced to 0.139 and 0.141 eV due to the guanidine substitution (Table S1). This corresponds to the higher chemical reactivity of the newly designed guanidine analogues than the original inhibitors (Table 1).

Based on the docking studies and DFT studies’ encouraging results, all the four G9a–inhibitor complexes (BIX-01294, UNC0638, and their guanidine analogues) were subjected to molecular dynamics simulation for 1 μs each. Root-mean-square deviation (rmsd) plots of the complexes showed that the complexes were well stabilized throughout the simulation period. The rmsd value of protein bound with BIX-01294 and BIX-01294 guanidine analogues was well maintained below 2.5 Å throughout the simulation period. Similarly, a comparison of the rmsd of the ligands shows that the BIX-01294 guanidine analogue maintained a lower rmsd than BIX-01294. Atomic flexibility of G9a residues shows reduced flexibility when bound with guanidine analogues, even in the terminal segments of G9a. Also, stabilized radius of gyration for the G9a protein was observed when the BIX-01294 guanidine analogue was bound (Figure S1).

Similar observations were evidenced in G9a complexed with UNC0638 and its guanidine analogue. The rmsd of G9a bound with UNC0638 and UNC0638 guanidine analogue was well maintained below 2.4 Å, reflecting the complexes’ stability. The flexibility of G9a residues illustrates a similar degree of freedom with the UNC0638 and guanidine analogue. However, the ligand rmsd of UNC0638 guanidine analogue fluctuates, suggesting that the active site’s ligand is flexible (Figure S2). These results suggest that G9a and the newly designed guanidine analogues form a stable complex during molecular dynamics (MD) simulations, similar to the original G9a inhibitors.

Further, from the simulation trajectories, frames were extracted at 100 ns intervals and overlaid to visualize the ligands’ conformations in the protein during the simulations. Unlike BIX-01294, the BIX-01294 guanidine analogue orientation was well maintained without many structural deviations (Figure S3). Guanidine substitution in BIX-01294 (Figure S3B) decreases the bicyclic ring movement in the active site, whereas the solvent-exposed region of the ligand still exhibits a higher degree of rotational freedom. The flexibility of UNC0638 was reduced (Figure S3C) compared to that of BIX-01294 (Figure S3A) due to the presence of lysine mimic substitution. The conformational stability of UNC0638 in the active site of G9a was slightly altered as a result of guanidine substitution (Figure S3D). However, both UNC0638 and the guanidine analogue were anchored to G9a through the stable lysine mimic side chain. The reduced conformational freedom of the BIX-01294 guanidine analogue may favor stable intermolecular interactions between the protein and the ligand.

Intermolecular H-bonds formed between G9a and the inhibitors during molecular dynamics simulations are depicted as running average plots in Figure S4. At some instants of the simulation, BIX-01294 formed a maximum of four hydrogen bonds and, on average, less than two H-bonds. Simultaneously, the guanidine analogue of BIX-01294 makes a maximum of nine H-bonds and is maintained on an average of six H-bonds. Even though UNC0638 shows more H-bonds in docking studies, only one hydrogen bond was observed in a major part of the MD trajectory. A maximum of three H-bonds were observed at only a few specific frames of the simulation. This evidences that the interactions observed with UNC0638 during molecular docking studies are not stable and lost during the molecular dynamics simulations. However, the UNC0638 guanidine analogue maintained on an average of 4–6 H-bonds at different periods of the simulation (Figure S4). Analysis of MD simulations suggests that guanidine substitution on BIX-01294 and UNC0638 increases the ligands’ H-bonding potential with G9a in the dynamic conformational states.

**Figure 4:**
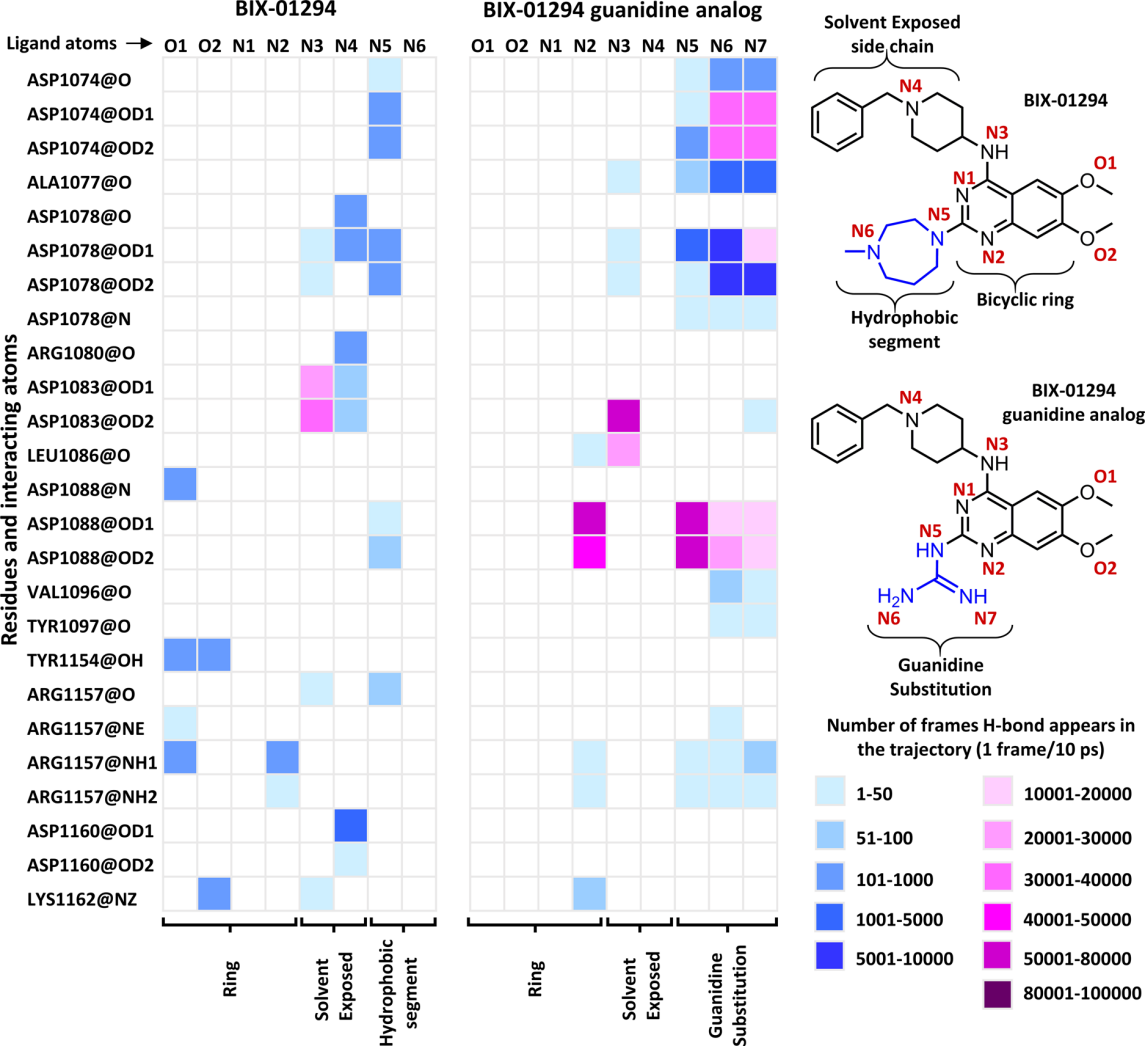
Number of frames H-bonds formed between G9a residues and the inhibitors (BIX-01294 and BIX-01294 guanidine analogue) during MD simulation.

**Figure 5:**
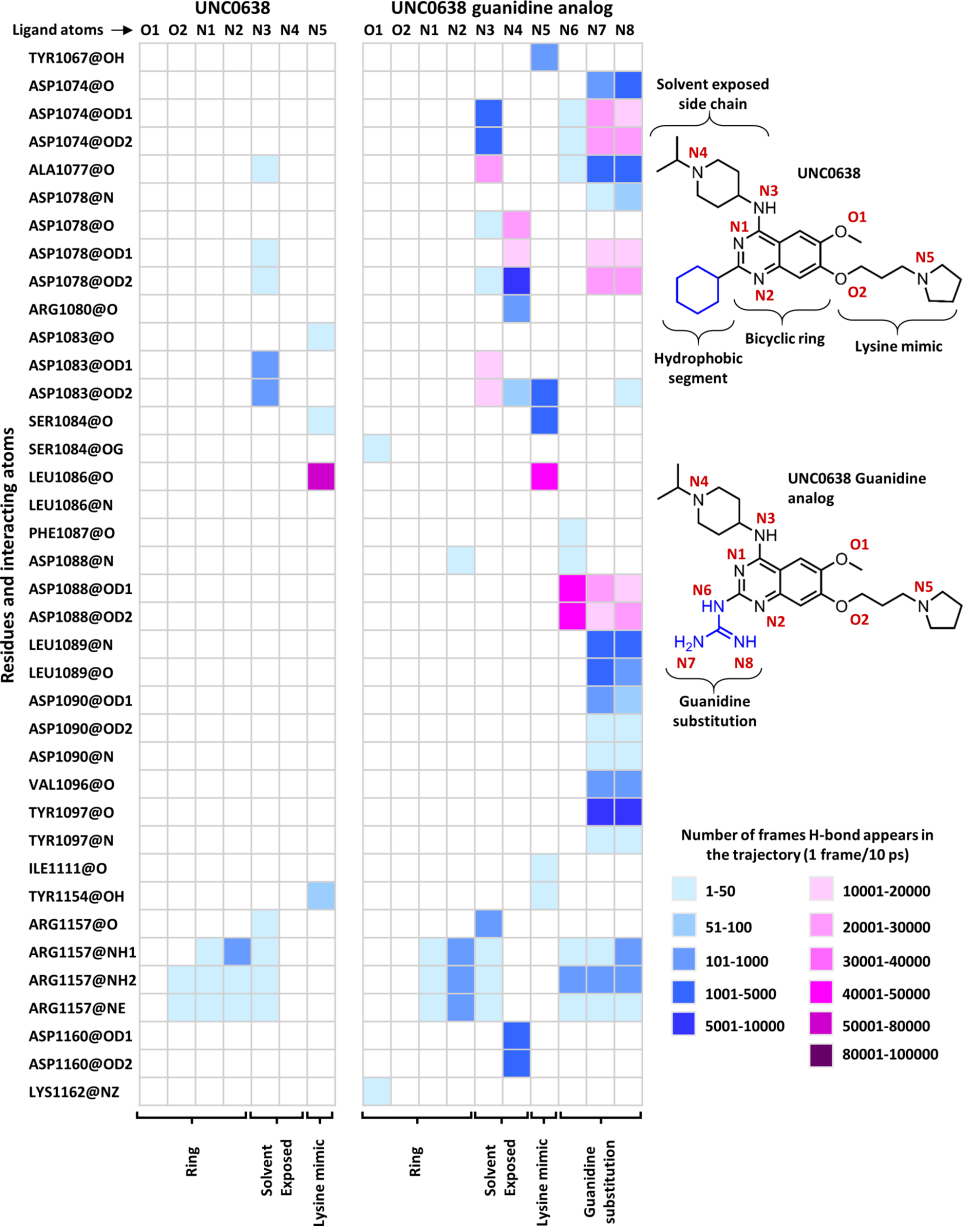
Number of frames H-bonds formed between G9a residues and the inhibitors (UNC0638 and UNC0638 guanidine analogue) during MD simulation.

Additionally, to investigate the ligand structural features responsible for the increase in H-bonds for the guanidine analogues, the inhibitor regions were segmented as bicyclic ring, hydrophobic segment, guanidine substitution, solvent-exposed chain, and lysine mimic side chain. The number of frames the H-bonds formed with the ligand segment’s polar atoms was calculated during the simulation and analyzed (Figures 4 and 5). The hydrophobic segment of BIX-01294 has two nitrogen atoms (N5 and N6) in the homopiperazine ring; the nitrogen atom (N5) placed near the bicyclic ring formed most of the H-bond interactions in this segment with Asp1074 and Asp1078. Substitution by the guanidine group at this segment made the guanidine nitrogen atoms (N5, N6, and N7) form H-bonds in more number of frames with the G9a residues, particularly with Asp1074, Asp1078, and Asp1088 (Figure 4). Furthermore, substitution by the guanidine moiety in BIX-01294 also increased the frequency of the H-bonds in other segments of the inhibitors through stabilizing the ligand conformation. The N2 atom of the bicyclic ring shows H-bonds with Asp1088 in more frames in the BIX-01294 guanidine analogue, which was not observed in BIX-01294.

Unlike BIX-01294, UNC0638 does not possess any polar atoms in its hydrophobic segment. Guanidine substitution at this position led to H-bonds between the guanidine nitrogens (N6, N7, and N8) and the residues Asp1074, Ala1077, Asp1078, Asp1088, Leu1089, Asp1090, Val1096, Tyr1097, and Arg1157 in more number of frames during the MD simulation (Figure 5). The bicyclic ring N3 made H-bond with a backbone of Ala1077 and the side-chain oxygen of Asp1083; the lysine mimic side-chain N5 made H-bond with Leu1086. Guanidine substitution in UNC0638 has resulted in stable interactions between the G9a and the ligand, as evidenced during MD simulations. Of particular interest is the specific H-bond formed between lysine mimic N5 and Leu1086 that appears for ∼42,439 frames (1 lakh frames were written) of the trajectory, which was not observed in UNC0638. This suggests that incorporating the guanidine group in the inhibitor to mimic Arg8 of H3 substrate peptide has considerably improved interaction in the lysine-binding tunnel of G9a. In contrast, the most notable H-bond interaction in UNC0638 is the N3 (solvent-exposed) with the residue Asp1083 and bicyclic ring N2 with the residue Arg1157.

The H-bonds formed during MD simulation for all the four ligands are depicted in Figure S5. The BIX-01294 guanidine analogue had H-bond interaction with Asp1074, Ala1077, Asp1078, Asp1083, Leu1086, and Asp1088; while, the UNC0638 guanidine analogue had H-bond interaction with Asp1074, Ala1077, Asp1078, Asp1083, Leu1086, Asp1088, Leu1089, Tyr1097, Arg1157, and Asp1160. It should be noted that based on the H3 substrate peptide and inhibitor binding mode analysis,^32^ we have analyzed and identified that compounds having favorable interaction with the G9a residues—Asp1074, Asp1083, Leu1086, Asp1088, Tyr1154, and Phe1158—possess stronger G9a inhibition. Out of the six residues, four residues (Asp1074, Asp1083, Leu1086, and Asp1088) made H-bond interaction with the newly designed guanidine analogues. Particularly, the three negatively charged aspartic acid residues of G9a—Asp1074, Asp1078, and Asp1088—exhibited more number of H-bonds; among these, interaction with Asp1088 was found to be the strongest. It should be noted that these aspartic acid residues are essential and complement the binding of the two functionally important positively charged residues Arg8 and Lys9 of the H3 substrate peptide, suggesting that the newly designed guanidine analogues can make interaction with the most important residues of G9a active site and would inhibit its function.

Next, binding free energy values of the ligands were calculated using two end-point energy calculation approaches, molecular mechanics Poisson–Boltzmann surface area (MMPBSA) and linear interaction energy (LIE) calculations. The MMPBSA method calculates the binding free energy using molecular mechanics with Poisson–Boltzmann and surface-area solvation.^36^ LIE estimates the binding free energy of the ligand by simulating the ligand in macromolecule and the free ligand in solution. In LIE, the free energy of the ligands was assumed to be proportional to the differences between the bound state and the unbound state van der Waals (vdW) and electrostatic energies.^37,38^ In MMPBSA calculations, BIX-01294 and UNC0638 show a binding energy of −33 kcal/mol. Whereas, the guanidine analogue of BIX-01294 and UNC0638 showed higher binding energies of −53 and −67 kcal/mol, respectively (Table 1). The results shows that the guanidine group’s introduction resulted in ∼twofold increased binding energy compared to the original ligands BIX-01294 and UNC0638. In the case of LIE calculations, BIX-01924 and UNC0638 showed energy values of −57.47 and −51.36 kcal/mol, respectively. The LIE of guanidine analogues of BIX-01294 and UNC0638 was calculated as −66.4265 and −117.2585 kcal/mol (Table 1), respectively. In both MMPBSA and LIE approaches, binding energy values are higher for the guanidine analogues than the known reported inhibitors. Moreover, the binding energies of the guanidine analogues to G9a were found to be higher than the calculated binding energies of the H3 substrate peptide (−51.67 kcal/mol).^32^

Further, steered molecular dynamics (SMD) simulations of the inhibitors and the newly designed analogues were performed to assess the difference in the dissociation force (rupture force) required to remove the ligands from the active site of G9a.^39^ Comparison of the known inhibitors, BIX-1294 and UNC0638, with their guanidine analogues shows that the addition of the guanidine group drastically increases the rupture force of the analogues (Table 1). The presence of the lysine mimic in UNC0638 did not affect the rupture force, as both BIX-1294 and UNC0638 require a similar level of rupture force. However, the guanidine group’s introduction to both BIX-1294 and UNC0638 increased the rupture force, which is more evident in the case of the UNC0638 guanidine analogue. This shows that lysine and guanidine mimic groups would make the ligand dissociation from the target more difficult. Moreover, the rupture force for the ligands corroborates well with the binding affinity calculation from MD simulations.

**Figure 6:**
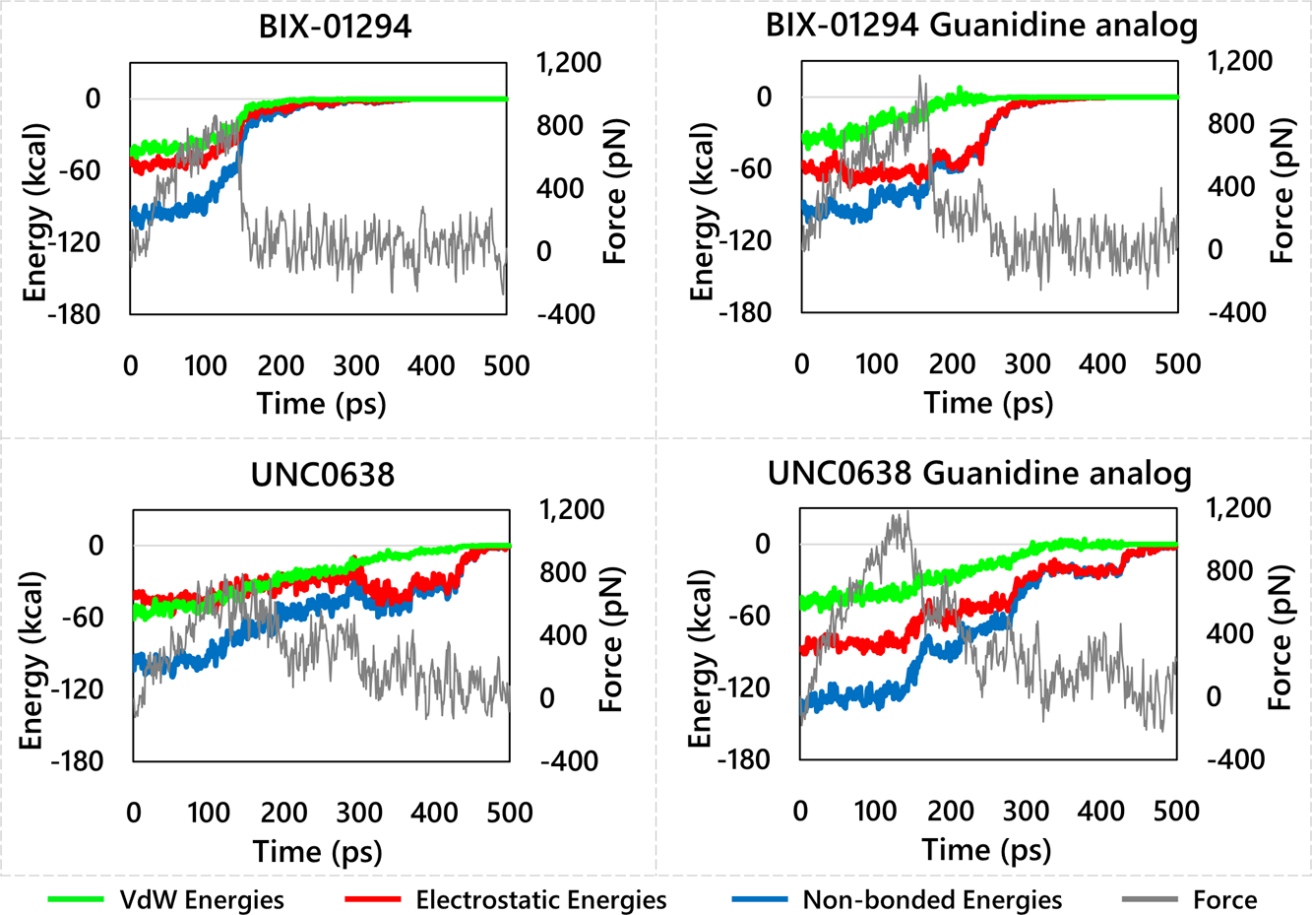
Results from SMD simulations of G9a inhibitors and their guanidine analogues. Each plot shows the nonbonded interaction energy terms (in kcal) between G9a–inhibitor complexes and the force (in pN) required during dissociation of the inhibitors and the guanidine analogues from the G9a (Video files S1–S4 of the representative SMD runs are given in the Supporting Information plots for multiple runs are given in Figure S6 of the Supporting Information).

The nonbonded interaction energies (vdW and electrostatic terms) during the dissociation phase of the ligand in the SMD experiment were plotted and are shown in Figure 6 (also in Figure S6). The strength of these interactions and the duration of these interactions were analyzed during the ligand dissociation from the G9a. Calculated nonbonded energies were similar for the known inhibitors BIX-01294 and UNC0638 at the initial bound conformation. Later, during the dissociation phase, BIX-01294 loses the interactions with G9a in a shorter time in comparison with UNC0638. The lysine mimic group, which is positioned in the lysine tunnel, assists UNC0638 to sustain the interactions for a longer time during dissociation. Particularly, the electrostatic interaction was sustained for a longer duration for UNC0638 compared to that of BIX-01294.

Comparison of BIX-01294 and BIX-01294 guanidine analogues shows only a slight increase in the nonbonded interaction energies at the bound initial conformation. However, the BIX-01294 guanidine analogue maintained the interactions, particularly the electrostatic interaction, with G9a for a longer time than BIX-0124. This resulted in longer time duration for the BIX-01294 guanidine analogue unbinding from G9a. Moreover, lysine and arginine mimic together in the UNC0638 guanidine analogue, which drastically increased the inhibitor’s nonbonded interaction energy and extended the interactions for a long time similar to that of UNC0638. The stronger and longer interaction of the UNC0638 guanidine analogue with G9a is responsible for the higher rupture force required to remove the ligand from G9a. Further, the H-bond frequency during the ligand dissociation simulations shows the interaction frequency of the newly designed analogues’ guanidine moiety with three residues Asp1074, Asp1078, and Asp1088 higher than any other H-bond formed during the ligand dissociation (Figures S7 and S8). Interactions with these three residues account for the higher nonbonded energy and rupture force of the newly designed analogues of the known inhibitors. These results corroborate well with the binding affinity calculation from MD simulations. In MD simulations also, these three residues Asp1074, Asp1078 and Asp1088 showed more frequent H-bonds with the newly designed guanidine analogues and improved the binding strength of the guanidine analogues to G9a.

In addition, the potential mean force (PMF) of the ligand dissociation was calculated from one of the representative SMD run, which showed good convergence in the mean rupture force and interaction energy profile. The PMF yields the free energy as the difference between the highest and the lowest points of the PMF curve.^40,41^ There is a significant difference in PMF between the compounds and their guanidine analogues (Figure S9). A positive PMF curve indicates that it is energetically favorable for the ligand to be bound with its receptors. Comparison of the PMF profile of the known inhibitors and their guanidine inhibitors shows that the guanidine analogues favor better binding to G9a than that of the known inhibitors. The PMF of BIX-01294 guanidine analogue (148.23 kcal/mol) is significantly higher than that of the BIX-01294 (101.49 kcal/mol). Having substituted with both lysine and arginine mimics, the UNC0638 guanidine analogue (345.53 kcal/mol) exhibits a large PMF than the known inhibitor UNC0638 (253.041 kcal/mol) and BIX-01294 and BIX-01294 guanidine analogues.

## 3 Conclusions

One of the techniques used in computer-aided drug design is a structure-based drug design (SBDD). SBDD is a promising approach since it helps discover molecules based on the protein–substrate, protein–ligand, or protein–protein interactions. Using SBDD, new ligands could be designed or optimized so that there is a shape, size, or charge complementarity between the target protein and the ligand molecules.^42−44^ Using the principles of SBDD, new G9a inhibitors are designed based on the G9a–H3 substrate peptide and G9a–inhibitor interaction information.

In our previous publication, using molecular dynamics simulations, we have shown the structural essentiality of Arg8 for efficient binding of the H3 substrate peptide to G9a.^32^ Based on the observations, here, the guanidine group (side chain of Arg) was introduced to known G9a inhibitors (BIX-01294 and UNC0638) to mimic the Arg8 of the H3 substrate peptide. An appropriate position for introducing the guanidine group in the G9a inhibitor was chosen by overlaying the inhibitor and the H3 substrate peptide binding conformation in G9a. Based on the structural insights, the guanidine group was introduced at the 2-position of quinazoline rings of BIX-01294 and UNC0638.

The newly designed BIX-01294 and UNC0638 guanidine analogues were evaluated for their G9a binding ability using docking, DFT, and (steered) molecular dynamics simulation experiments. MMPBSA- and LIE-based binding energy calculation shows a significant improvement in binding for the guanidine analogues compared to the original ligands. Further, dissecting the G9a–ligand interactions shows that guanidine substitution improves the binding potential of the ligands by making H-bonds with three of the negatively charged residues Asp1074, Asp1078, and Asp1088 in the active site of G9a. In particular, the guanidine analogue of UNC0638 shows consistent and stable H-bonds with Leu1086 in the lysine tunnel of G9a. Additionally, guanidine substitutions increased the dissociation force required to remove the ligand from the target in SMD simulations and maintained more vital electrostatic interaction with Asp1074, Asp1078, and Asp1088 for a longer duration in the G9a binding site. PMF and the free energy of the ligand unbinding process are also higher for the guanidine analogues. Moreover, the *in silico* ADME profile (Table S2) suggests that the guanidine analogues would be advantageous.^45^ Based on the computational evaluation, we suggest that BIX-01294 and UNC0638 guanidine analogues are potential lead molecules that could be synthesized and tested for G9a inhibition.

## 4 Computational Methods

### 4.1 Model Building and Molecular Docking

The cocrystal structure of G9a in complex with UNC0638 (PDB ID: 3RJW) was retrieved from the protein data bank. The structure was prepared, optimized, and minimized using the protein preparation wizard of Schrodinger Maestro 11.5.^46^ The PROPKA package was used to assign the protein residues’ protonation states at neutral pH.^47^ Since the calculated p*K*_a_ values at three different pH (6.5, 7, and 7.5) values do not alter the protonation states of the active site residues, protonation states of the residues were assigned at a pH of 7.4. Further, p*K*_a_ values and protonation states of the active site residues can be studied in detail either by computations or by experimental studies as reported earlier.^48−50^ The active site was defined by the bound ligand UNC0638 for docking of UNC0638 and its guanidine analogue. Whereas in the case of BIX-01294, the cocrystallized structure is available only with GLP (G9a-like protein), an isoform of G9a sharing identical catalytic-site residues and inhibitor binding mode.^51,52^ Since earlier studies reported no structural differences between the G9a and GLP inhibitor binding modes, GLP-bound conformation of BIX-01294 (PDB ID: 3FPD) was adapted to form the complex with the G9a protein (from PDB ID: 3RJW) through structural superimposition. The active site was set around the adapted conformation of BIX-01294 for docking of BIX-01294 and BIX-01294 guanidine analogue. Conformations and ionization states of ligands were prepared at pH 7 ± 2 using the ligprep module and docked into the prepared G9a structures using the extra precision mode of the glide module available on the Maestro 11.5 platform from Schrodinger.^53^ Inclusion of the originally known cocrystal ligand UNC0638 serves as a validity measure of the docking procedure. Redocking showed that the predicted conformation and cocrystal conformation of UNC0638 has an rmsd of 0.145 Å (Figure S10). Induced fit docking of the compounds were performed on the defined grid using glide with 5 Å residue flexibility for the active-site residues.

### 4.2 DFT Calculations

The docked orientation of the inhibitors and designed guanidine analogues was used for DFT calculations. Molecular features such as frontier molecular orbitals—HOMO and LUMO and electron density were calculated using the functional B3LYP with 6-3IG* basic set.^54^ These DFT studies were performed to calculate the energy gap between molecular orbitals. All calculations were performed using the Jaguar module available on the Maestro 11.5 platform from Schrodinger.^53,55^

### 4.3 MD Simulation

MD simulations of the protein–ligand complexes were carried out with Amber16.^56^ Amber14SB force field parameters for a protein molecule and ligand parameters were generated using the Antechamber program from AmberTools v17. Restrained electrostatic potential (RESP) partial charges of the ligand atoms were calculated at the HF level with a 6-31G*(1d) basis set using Gaussian v03.^57^ Standard protonation states for the residues were defined by the force field using Amber tools v17. Crystallographic water molecules located around 5 Å of the ligand and the cofactor were included in the simulation system. TIP3P water molecules were filled around the protein in a rectangular box with dimensions of 85.2′ 73.3′ 77.35 Å (i.e., 10 Å from the protein’s edges). Bulk water molecules were replaced during neutralization, and 0.150 mM concentration NaCl was added to the system. The prepared complexes were energy-minimized in two steps (first 5000 steps followed by 10,000 steps) and then gradually heated for 50 ps, and the complex equilibrated for 500 ps. Finally, the complex was subjected to a production run for 1 μs using the AMBER v16 package. The coordinates were written for every 10 picoseconds (1 frame/10 ps).^58^

### 4.4 Binding Energy Calculations

The binding free energy of the compounds was calculated using MMPBSA and LIE methods. The MMPBSA.py program^59^ of AMBER was used to calculate the binding energy from the 2000 frames extracted from the microsecond simulation trajectory. For LIE calculations, the ligand–protein complexes were simulated for a period of 50 ns and ligand alone in water for a period of 20 ns. From each protein–ligand complex and ligand trajectory, 500 frames were extracted and used for LIE estimation. LIE was calculated using CaFE v1.0 (VMD plugin) with default vdW and electrostatic scaling parameters (α = 0.18, β = 0.5, and γ = 0).^60^

### 4.5 SMD Simulations and PMF Calculation

All SMD simulations were performed in NAMD v2.12.^61^ From the 1 μs Amber MD trajectory, three frames from 800th, 900th, and 1000th ns were extracted and used for SMD. SMD simulations were performed thrice for each of the extracted frames. Pulling parameters, particularly the spring constant and the pulling velocity, were set to 5 kcal/mol·Å^2^ and 0.005 Å/ps, respectively. The ligands were pulled to a distance of 25 Å from their initial orientation so that the protein–ligand interactions will be lost completely. A detailed SMD protocol is reported elsewhere.^58^ The highest force recorded in multiple SMD simulations for three different frames was averaged to calculate the mean rupture force. The rupture force was evaluated in the pulling direction, and the NAMD Energy plugin was used to generate the interaction energy plot during dissociation simulations.^61^

Further, to calculate free energy of the ligand unbinding process, representative frames from the SMD simulations were selected for umbrella sampling (see Figure S6, Run-1 from 1000 ns for BIX-01294, BIX-01294 guanidine analogue and UNC0638. Run-2 from 1000 ns for UNC0638 guanidine analogue). From these dissociation trajectories, windows were extracted between the bound state and the state in which the ligand completely dissociated (12 windows for BIX-01294, 17 windows for BIX-01294 guanidine analogue, 24 windows for UNC0638, and 25 windows for the UNC0638 guanidine analogue). Umbrella sampling windows were selected with the stride of 1 Å distance for calculating PMF, and the simulation time was set as 1 ns. The PMF profile of the ligand dissociation process was analyzed with weighted histogram analysis and the bootstrap analysis method.^62,63^

## Supporting Information Available

Molecular orbital and physiochemical properties of inhibitors, rmsd, fluctuation and H-bond plots/frequencies from MD simulations; and H-bond frequency heat maps from SMD and PMF plots (PDF)
Video S1: G9a - BIX-01924 – SMD (representative) (MP4)
Video S2: G9a - BIX-01924_guanidine analog − SMD (representative) (MP4)
Video S3: G9a - UNC0638 − SMD (representative) (MP4)
Video S4: G9a - UNC0638_guanidine analog − SMD (representative) (MP4)
PDB-S1: G9a - BIX-01924 docked complex (PDB)
PDB-S2: G9a - BIX-01924_guanidine analog docked complex (PDB)
PDB-S3: G9a - UNC0638 docked complex (PDB)
PDB-S4: G9a - UNC0638_guanidine analog docked complex (PDB)

## Supplementary Material

ao0c04710_si_005.mp4

ao0c04710_si_006.pdb

ao0c04710_si_007.pdb

ao0c04710_si_008.pdb

ao0c04710_si_009.pdb

## References

[ref1] Tachibana M.; Sugimoto K.; Fukushima T.; Shinkai Y. SET Domain-Containing Protein, G9a, Is a Novel Lysine-Preferring Mammalian Histone Methyltransferase with Hyperactivity and Specific Selectivity to Lysines 9 and 27 of Histone H3. J. Biol. Chem. 2001, 276, 25309–25317. 10.1074/jbc.m101914200. 11316813

[ref2] Ogawa H.; Ishiguro K. I.; Gaubatz S.; Livingston D. M.; Nakatani Y. A Complex with Chromatin Modifiers That Occupies E2f- and Myc-Responsive Genes in G0 Cells. Science 2002, 296, 1132–1136. 10.1126/science.1069861. 12004135

[ref3] Shankar S. R.; Bahirvani A. G.; Rao V. K.; Bharathy N.; Ow J. R.; Taneja R. G9a, a Multipotent Regulator of Gene Expression. Epigenetics 2013, 8, 16–22. 10.4161/epi.23331. 23257913 PMC3549875

[ref4] Tachibana M.; Ueda J.; Fukuda M.; Takeda N.; Ohta T.; Iwanari H.; Sakihama T.; Kodama T.; Hamakubo T.; Shinkai Y. Histone Methyltransferases G9a and GLP Form Heteromeric Complexes and Are Both Crucial for Methylation of Euchromatin at H3-K9. Genes Dev. 2005, 19, 815–826. 10.1101/gad.1284005. 15774718 PMC1074319

[ref5] Peters A. H. F. M.; Kubicek S.; Mechtler K.; O’Sullivan R. J.; Derijck A. A. H. A.; Perez-Burgos L.; Kohlmaier A.; Opravil S.; Tachibana M.; Shinkai Y.; et al. Partitioning and Plasticity of Repressive Histone Methylation States in Mammalian Chromatin. Mol. Cell 2003, 12, 1577–1589. 10.1016/s1097-2765(03)00477-5. 14690609

[ref6] Rice J. C.; Briggs S. D.; Ueberheide B.; Barber C. M.; Shabanowitz J.; Hunt D. F.; Shinkai Y.; Allis C. D. Histone Methyltransferases Direct Different Degrees of Methylation to Define Distinct Chromatin Domains. Mol. Cell 2003, 12, 1591–1598. 10.1016/s1097-2765(03)00479-9. 14690610

[ref7] Imai K.; Togami H.; Okamoto T. Involvement of Histone H3 Lysine 9 (H3K9) Methyltransferase G9a in the Maintenance of HIV-1 Latency and Its Reactivation by BIX01294. J. Biol. Chem. 2010, 285, 16538–16545. 10.1074/jbc.m110.103531. 20335163 PMC2878073

[ref8] Nguyen K.; Das B.; Dobrowolski C.; Karn J. Multiple Histone Lysine Methyltransferases Are Required for the Establishment and Maintenance of HIV-1 Latency. mBio 2017, 8, e00133 10.1128/mbio.00133-17. 28246360 PMC5347344

[ref9] Shi Y.; Desponts C.; Do J. T.; Hahm H. S.; Schöler H. R.; Ding S. Induction of Pluripotent Stem Cells from Mouse Embryonic Fibroblasts by Oct4 and Klf4 with Small-Molecule Compounds. Cell Stem Cell 2008, 3, 568–574. 10.1016/j.stem.2008.10.004. 18983970

[ref10] Epsztejn-Litman S.; Feldman N.; Abu-Remaileh M.; Shufaro Y.; Gerson A.; Ueda J.; Deplus R.; Fuks F.; Shinkai Y.; Cedar H.; et al. De Novo DNA Methylation Promoted by G9a Prevents Reprogramming of Embryonically Silenced Genes. Nat. Struct. Mol. Biol. 2008, 15, 1176–1183. 10.1038/nsmb.1476. 18953337 PMC2581722

[ref11] Thienpont B.; Aronsen J. M.; Robinson E. L.; Okkenhaug H.; Loche E.; Ferrini A.; Brien P.; Alkass K.; Tomasso A.; Agrawal A.; et al. The H3K9 Dimethyltransferases EHMT1/2 Protect against Pathological Cardiac Hypertrophy. J. Clin. Invest. 2017, 127, 335–348. 10.1172/JCI88353. 27893464 PMC5199699

[ref12] Tarantini S.; Valcarcel-Ares N. M.; Yabluchanskiy A.; Fulop G. A.; Hertelendy P.; Gautam T.; Farkas E.; Perz A.; Rabinovitch P. S.; Sonntag W. E.; et al. Treatment with the Mitochondrial-Targeted Antioxidant Peptide SS-31 Rescues Neurovascular Coupling Responses and Cerebrovascular Endothelial Function and Improves Cognition in Aged Mice. Aging Cell 2018, 17, e12731 10.1111/acel.12731. PMC5847870 29405550

[ref13] Vilhais-Neto G. C.; Fournier M.; Plassat J. L.; Sardiu M. E.; Saraf A.; Garnier J. M.; Maruhashi M.; Florens L.; Washburn M. P.; Pourquié O. The WHHERE Coactivator Complex Is Required for Retinoic Acid-Dependent Regulation of Embryonic Symmetry. Nat. Commun. 2017, 8, 728 10.1038/s41467-017-00593-6. 28959017 PMC5620087

[ref14] Luo C.-W.; Wang J.-Y.; Hung W.-C.; Peng G.; Tsai Y.-L.; Chang T.-M.; Chai C.-Y.; Lin C.-H.; Pan M.-R. G9a Governs Colon Cancer Stem Cell Phenotype and Chemoradioresistance through PP2A-RPA Axis-Mediated DNA Damage Response. Radiother. Oncol. 2017, 124, 395–402. 10.1016/j.radonc.2017.03.002. 28351524

[ref15] Benevento M.; van de Molengraft M.; van Westen R.; van Bokhoven H.; Nadif Kasri N. The Role of Chromatin Repressive Marks in Cognition and Disease: A Focus on the Repressive Complex GLP/G9a. Neurobiol. Learn. Mem. 2015, 124, 88–96. 10.1016/j.nlm.2015.06.013. 26143996

[ref16] Greiner D.; Bonaldi T.; Eskeland R.; Roemer E.; Imhof A. Identification of a Specific Inhibitor of the Histone Methyltransferase SU(VAR)3-9. Nat. Chem. Biol. 2005, 1, 143–145. 10.1038/nchembio721. 16408017

[ref17] Huang S. Histone Methyltransferases, Diet Nutrients and Tumour Suppressors. Nat. Rev. Cancer 2002, 2, 469–476. 10.1038/nrc819. 12189389

[ref18] Kubicek S.; O’Sullivan R. J.; August E. M.; Hickey E. R.; Zhang Q.; Teodoro M. L.; Rea S.; Mechtler K.; Kowalski J. A.; Homon C. A.; et al. Reversal of H3K9me2 by a Small-Molecule Inhibitor for the G9a Histone Methyltransferase. Mol. Cell 2007, 25, 473–481. 10.1016/j.molcel.2007.01.017. 17289593

[ref19] Chang Y.; Ganesh T.; Horton J. R.; Spannhoff A.; Liu J.; Sun A.; Zhang X.; Bedford M. T.; Shinkai Y.; Snyder J. P.; et al. Adding a Lysine Mimic in the Design of Potent Inhibitors of Histone Lysine Methyltransferases. J. Mol. Biol. 2010, 400, 1–7. 10.1016/j.jmb.2010.04.048. 20434463 PMC2895764

[ref20] Liu F.; Chen X.; Allali-Hassani A.; Quinn A. M.; Wigle T. J.; Wasney G. a.; Dong A.; Senisterra G.; Chau I.; Siarheyeva A.; et al. Protein Lysine Methyltransferase G9a Inhibitors: Design, Synthesis, and Structure Activity Relationships of 2,4-Diamino-7-Aminoalkoxy-Quinazolines. J. Med. Chem. 2010, 53, 5844–5857. 10.1021/jm100478y. 20614940 PMC2920043

[ref21] Liu F.; Chen X.; Allali-Hassani A.; Quinn A. M.; Wasney G. A.; Dong A.; Barsyte D.; Kozieradzki I.; Senisterra G.; Chau I.; et al. Discovery of a 2,4-Diamino-7-Aminoalkoxyquinazoline as a Potent and Selective Inhibitor of Histone Lysine Methyltransferase G9a. J. Med. Chem. 2009, 52, 7950–7953. 10.1021/jm901543m. 19891491 PMC2825141

[ref22] Vedadi M.; Barsyte-Lovejoy D.; Liu F.; Rival-Gervier S.; Allali-Hassani A.; Labrie V.; Wigle T. J.; Dimaggio P. A.; Wasney G. A.; Siarheyeva A.; et al. A Chemical Probe Selectively Inhibits G9a and GLP Methyltransferase Activity in Cells. Nat. Chem. Biol. 2011, 7, 566–574. 10.1038/nchembio.599. 21743462 PMC3184254

[ref23] Liu F.; Barsyte-Lovejoy D.; Allali-Hassani A.; He Y.; Herold J. M.; Chen X.; Yates C. M.; Frye S. V.; Brown P. J.; Huang J.; Vedadi M.; Arrowsmith C. H.; Jin J. Optimization of Cellular Activity of G9a Inhibitors 7-Aminoalkoxy-Quinazolines. J. Med. Chem. 2011, 54, 6139–6150. 10.1021/jm200903z. 21780790 PMC3171737

[ref24] Liu F.; Barsyte-Lovejoy D.; Li F.; Xiong Y.; Korboukh V.; Huang X.-P.; Allali-Hassani A.; Janzen W. P.; Roth B. L.; Frye S. V.; et al. Discovery of an in Vivo Chemical Probe of the Lysine Methyltransferases G9a and GLP. J. Med. Chem. 2013, 56, 8931–8942. 10.1021/jm401480r. 24102134 PMC3880643

[ref25] Sweis R. F.; Pliushchev M.; Brown P. J.; Guo J.; Li F.; Maag D.; Petros A. M.; Soni N. B.; Tse C.; Vedadi M.; et al. Discovery and Development of Potent and Selective Inhibitors of Histone Methyltransferase G9a. ACS Med. Chem. Lett. 2014, 5, 205–209. 10.1021/ml400496h. 24900801 PMC4027767

[ref26] San José-Enériz E.; Agirre X.; Rabal O.; Vilas-Zornoza A.; Sanchez-Arias JA; Miranda E.; Ugarte A.; Roa S.; Paiva B.; Estella-Hermoso de Mendoza A.; et al. Discovery of First-in-Class Reversible Dual Small Molecule Inhibitors against G9a and DNMTs in Hematological Malignancies. Nat. Commun. 2017, 8, 15424 10.1038/ncomms15424. 28548080 PMC5458547

[ref27] Charles M. R. C.; Dhayalan A.; Hsieh H. P.; Coumar M. S. Insights for the Design of Protein Lysine Methyltransferase G9a Inhibitors. Future Medicinal Chemistry; Future Medicine Ltd., May 29, 2019; pp 993–1014. 10.4155/fmc-2018-0396 31141392

[ref28] Juárez-Salcedo L. M.; Desai V.; Dalia S. Venetoclax: Evidence to Date and Clinical Potential. Drugs Context 2019, 8, 212574 10.7573/dic.212574. 31645879 PMC6788387

[ref29] Kim A.; Cohen M. S. The Discovery of Vemurafenib for the Treatment of BRAF-Mutated Metastatic Melanoma. Expet Opin. Drug Discov. 2016, 11, 907–916. 10.1080/17460441.2016.1201057. PMC5443413 27327499

[ref30] Williams S. P.; Kuyper L. F.; Pearce K. H. Recent Applications of Protein Crystallography and Structure-Guided Drug Design. Current Opinion in Chemical Biology; Elsevier Current Trends, August 1, 2005; pp 371–80. 10.1016/j.cbpa.2005.06.007 16006182

[ref31] Rathert P.; Dhayalan A.; Murakami M.; Zhang X.; Tamas R.; Jurkowska R.; Komatsu Y.; Shinkai Y.; Cheng X.; Jeltsch A. Protein Lysine Methyltransferase G9a Acts on Non-Histone Targets. Nat. Chem. Biol. 2008, 4, 344–346. 10.1038/nchembio.88. 18438403 PMC2696268

[ref32] Ramya Chandar Charles M.; Hsieh H.-P.; Selvaraj Coumar M. Delineating the Active Site Architecture of G9a Lysine Methyltransferase through Substrate and Inhibitor Binding Mode Analysis: A Molecular Dynamics Study. J. Biomol. Struct. Dyn. 2018, 37, 2581–2592. 10.1080/07391102.2018.1491422. 30047835

[ref33] Nguyen K. T.; Li F.; Poda G.; Smil D.; Vedadi M.; Schapira M. Strategy to Target the Substrate Binding Site of SET Domain Protein Methyltransferases. J. Chem. Inf. Model. 2013, 53, 681–691. 10.1021/ci300596x. 23410263

[ref34] Sakkiah S.; Lee K. W. Pharmacophore-Based Virtual Screening and Density Functional Theory Approach to Identifying Novel Butyrylcholinesterase Inhibitors. Acta Pharmacol. Sin. 2012, 33, 964–978. 10.1038/aps.2012.21. 22684028 PMC4077067

[ref35] Queiroz A. N.; Gomes B. A. Q.; Moraes W. M. Jr.; Borges R. S. A theoretical antioxidant pharmacophore for resveratrol. Eur. J. Med. Chem. 2009, 44, 1644–1649. 10.1016/j.ejmech.2008.09.023. 18976835

[ref36] Kollman P. A.; Massova I.; Reyes C.; Kuhn B.; Huo S.; Chong L.; Lee M.; Lee T.; Duan Y.; Wang W.; et al. Calculating Structures and Free Energies of Complex Molecules: Combining Molecular Mechanics and Continuum Models. Acc. Chem. Res. 2000, 33, 889–897. 10.1021/ar000033j. 11123888

[ref37] Hansson T.; Marelius J.; AÅqvist J. Ligand Binding Affinity Prediction by Linear Interaction Energy Methods. J. Comput. Aided Mol. Des. 1998, 12, 27–35. 10.1023/a:1007930623000. 9570087

[ref38] AÅqvist J.; Medina C.; Samuelsson J.-E. A New Method for Predicting Binding Affinity in Computer-Aided Drug Design. Protein Eng. Des. Sel. 1994, 7, 385–391. 10.1093/protein/7.3.385. 8177887

[ref39] Do P.-C.; Lee E. H.; Le L. Steered Molecular Dynamics Simulation in Rational Drug Design. J. Chem. Inf. Model. 2018, 58, 1473–1482. 10.1021/acs.jcim.8b00261. 29975531

[ref40] Doudou S.; Burton N. A.; Henchman R. H. Standard Free Energy of Binding from a One-Dimensional Potential of Mean Force. J. Chem. Theory Comput. 2009, 5, 909–918. 10.1021/ct8002354. 26609600

[ref41] Branduardi D.; Gervasio F. L.; Parrinello M. From A to B in Free Energy Space. J. Chem. Phys. 2007, 126, 054103 10.1063/1.2432340. 17302470

[ref42] Anderson A. C. The Process of Structure-Based Drug Design. Chem. Biol. 2003, 10, 787–797. 10.1016/j.chembiol.2003.09.002. 14522049

[ref43] Nero T. L.; Parker M. W.; Morton C. J. Protein Structure and Computational Drug Discovery. Biochem. Soc. Trans. 2018, 46, 1367–1379. 10.1042/bst20180202. 30242117

[ref44] Arkin M. R.; Tang Y.; Wells J. A. Small-Molecule Inhibitors of Protein-Protein Interactions: Progressing toward the Reality. Chemistry and Biology; Cell Press, 2014; pp 1102–1114 10.1016/j.chembiol.2014.09.001 PMC4179228 25237857

[ref45] Daina A.; Michielin O.; Zoete V. SwissADME: A Free Web Tool to Evaluate Pharmacokinetics, Drug-Likeness and Medicinal Chemistry Friendliness of Small Molecules. Sci. Rep. 2017, 7, 42717 10.1038/srep42717. 28256516 PMC5335600

[ref46] Schrödinger Inc. LigPrep|Schrödinger. Schrödinger Release 2018-1, 2018.

[ref47] Olsson M. H. M.; Søndergaard C. R.; Rostkowski M.; Jensen J. H. PROPKA3: Consistent Treatment of Internal and Surface Residues in Empirical p K a Predictions. J. Chem. Theory Comput. 2011, 7, 525–537. 10.1021/ct100578z. 26596171

[ref48] Czerwinski R. M.; Harris T. K.; Massiah M. A.; Mildvan A. S.; Whitman C. P. The Structural Basis for the Perturbed PKa of the Catalytic Base in 4-Oxalocrotonate Tautomerase: Kinetic and Structural Effects of Mutations of Phe-50. Biochemistry 2001, 40, 1984–1995. 10.1021/bi0024714. 11329265

[ref49] Repič M.; Purg M.; Vianello R.; Mavri J. Examining Electrostatic Preorganization in Monoamine Oxidases A and B by Structural Comparison and p Ka Calculations. J. Phys. Chem. B 2014, 118, 4326–32. 10.1021/jp500795p. 24678966

[ref50] Sham Y. Y.; Chu Z. T.; Warshel A. Consistent Calculations of PKa’s of Ionizable Residues in Proteins: Semi-Microscopic and Microscopic Approaches. J. Phys. Chem. B 1997, 101, 4458–4472. 10.1021/jp963412w.

[ref51] Xiong Y.; Li F.; Babault N.; Wu H.; Dong A.; Zeng H.; Chen X.; Arrowsmith C. H.; Brown P. J.; Liu J.; et al. Structure-Activity Relationship Studies of G9a-like Protein (GLP) Inhibitors. Bioorg. Med. Chem. 2017, 25, 4414–4423. 10.1016/j.bmc.2017.06.021. 28662962 PMC5562403

[ref52] Xiong Y.; Li F.; Babault N.; Dong A.; Zeng H.; Wu H.; Chen X.; Arrowsmith C. H.; Brown P. J.; Liu J.; et al. Discovery of Potent and Selective Inhibitors for G9a-Like Protein (GLP) Lysine Methyltransferase. J. Med. Chem. 2017, 60, 1876–1891. 10.1021/acs.jmedchem.6b01645. 28135087 PMC5352984

[ref53] Schrödinger Inc. Maestro|Schrödinger. Schrödinger Release 2018-1, 2018.

[ref54] Gill P. M. W.; Johnson B. G.; Pople J. A.; Frisch M. J. The Performance of the Becke-Lee-Yang-Parr (B-LYP) Density Functional Theory with Various Basis Sets. Chem. Phys. Lett. 1992, 197, 499–505. 10.1016/0009-2614(92)85807-m.

[ref55] Bochevarov A. D.; Harder E.; Hughes T. F.; Greenwood J. R.; Braden D. A.; Philipp D. M.; Rinaldo D.; Halls M. D.; Zhang J.; Friesner R. A. Jaguar: A High-Performance Quantum Chemistry Software Program with Strengths in Life and Materials Sciences. Int. J. Quantum Chem. 2013, 113, 2110–2142. 10.1002/qua.24481.

[ref56] Case D. A.; Cheatham T. E.; Darden T.; Gohlke H.; Luo R.; Merz K. M.; Onufriev A.; Simmerling C.; Wang B.; Woods R. J. The Amber Biomolecular Simulation Programs. J. Comput. Chem. 2005, 26, 1668–1688. 10.1002/jcc.20290. 16200636 PMC1989667

[ref57] Cornell W. D.; Cieplak P.; Bayly C. I.; Kollman P. A. Application of RESP Charges to Calculate Conformational Energies, Hydrogen Bond Energies, and Free Energies of Solvation. J. Am. Chem. Soc. 1993, 115, 9620–9631. 10.1021/ja00074a030.

[ref58] Charles M. R. C.; Mahesh A.; Lin S.-Y.; Hsieh H.-P.; Dhayalan A.; Coumar M. S. Identification of Novel Quinoline Inhibitor for EHMT2/G9a through Virtual Screening. Biochimie 2020, 168, 220–230. 10.1016/j.biochi.2019.11.006. 31756401

[ref59] Miller B. R.; McGee T. D.; Swails J. M.; Homeyer N.; Gohlke H.; Roitberg A. E. MMPBSA.Py: An Efficient Program for End-State Free Energy Calculations. J. Chem. Theory Comput. 2012, 8, 3314–3321. 10.1021/ct300418h. 26605738

[ref60] Liu H.; Hou T. CaFE: A Tool for Binding Affinity Prediction Using End-Point Free Energy Methods. Bioinformatics 2016, 32, 2216–2218. 10.1093/bioinformatics/btw215. 27153651

[ref61] Phillips J. C.; Braun R.; Wang W.; Gumbart J.; Tajkhorshid E.; Villa E.; Chipot C.; Skeel R. D.; Kalé L.; Schulten K. Scalable Molecular Dynamics with NAMD. J. Comput. Chem. 2005, 26, 1781–1802. 10.1002/jcc.20289. 16222654 PMC2486339

[ref62] Baştuğ T.; Chen P. C.; Patra S. M.; Kuyucak S. Potential of Mean Force Calculations of Ligand Binding to Ion Channels from Jarzynski’s Equality and Umbrella Sampling. J. Chem. Phys. 2008, 128, 155104 10.1063/1.2904461. 18433285

[ref63] Kumar S.; Rosenberg J. M.; Bouzida D.; Swendsen R. H.; Kollman P. A. THE Weighted Histogram Analysis Method for Free-energy Calculations on Biomolecules. I. The Method. J. Comput. Chem. 1992, 13, 1011–1021. 10.1002/jcc.540130812.

