# Supplementary material for: Mimicking H3 Substrate Arginine in the Design of G9a Lysine Methyltransferase Inhibitors for Cancer Therapy: A Computational Study for Structure-Based Drug Design
Source: ACS Omega. 2021 Feb 22;6(9):6100–11. doi: 10.1021/acsomega.0c04710 (PMC7948220; doi:10.1021/acsomega.0c04710)
Supplement: Supplementary file 1 — ao0c04710_si_001.pdf [file ao0c04710_si_001.pdf]

Supporting Information

Mimicking H3 substrate arginine in the design of G9a lysine methyltransferase inhibitors for cancer therapy: a computational study for structure-based drug design

M. Ramya Chandar Charles,[1] Mu-Chun Li,[2,3] Hsing-Pang Hsieh,[2,3,4*] Mohane Selvaraj Coumar.[1*]

[1]Centre for Bioinformatics, School of Life Sciences, Pondicherry University, Kalapet, Puducherry, 605014, India.

[2]Institute of Biotechnology and Pharmaceutical Research, National Health Research Institutes, 35 Keyan Road, Zhunan, Miaoli County 350, Taiwan, ROC.

[3]Department of Chemistry, National Tsing Hua University, No. 101, Section 2, Kuang-Fu Road, Hsinchu 300, Taiwan.

[4]Biomedical Translation Research Center, Academia Sinica, Taipei 115, Taiwan.

[*]Corresponding authors' e-mails: hphsieh@nhri.edu.tw & mohane@bicpu.edu.in

Table S1. Molecular orbitals energy gap of known G9a inhibitors and newly designed guanidine analogs.

Compound	Molecular Orbitals			
	E_{HOMO}	E_{LUMO}	ΔE (eV)	Electron density
BIX-01294	-0.174774	-0.029814	0.14496	551.126
BIX-01294 guanidine analog	-0.292966	-0.154000	0.138966	477.758
UNC0638	-0.497871	-0.349871	0.148000	594.143
UNC0638 guanidine analog	-0.330336	-0.189805	0.140531	550.799

Table S2. *In silico* ADME profile of the compounds included in the study.

Molecule	Lipinski rule (violations)	Liphophilicity (iLOGP)	Solubility (SILICOS-IT class)	BBB permeant	PAINS alerts
BIX-01294	0	4.67	Poorly soluble	Yes	0
BIX-01294 guanidine analog	0	3.42	Poorly soluble	No	0
UNC0638	1	5.67	Poorly soluble	Yes	0
UNC0638 guanidine analog	0	4.3	Moderately soluble	No	0

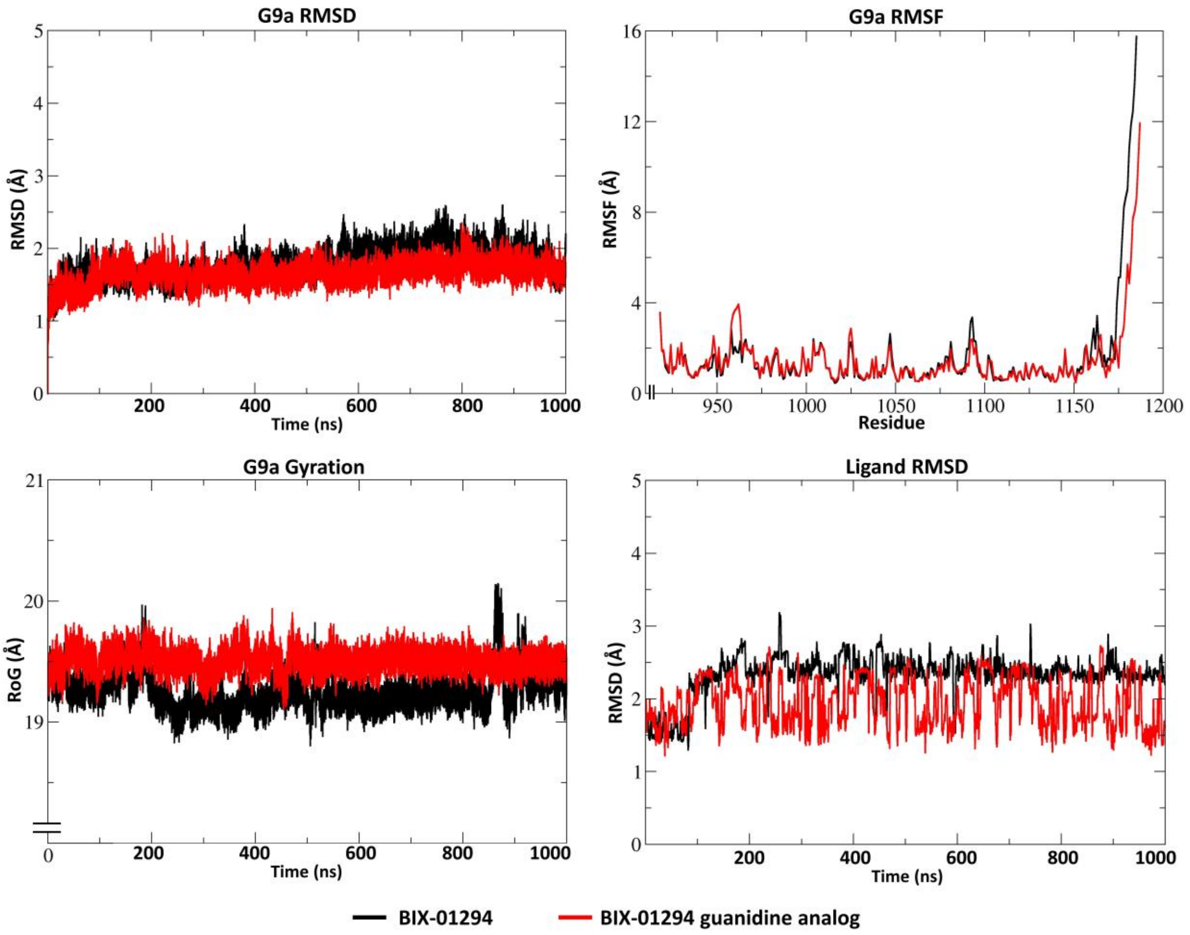

Figure S1. Root mean square deviation (RMSD), root mean square fluctuation (RMSF), radius of gyration and ligand RMSD plots of BIX-01294 and its guanidine analog from 1 µs MD simulation.

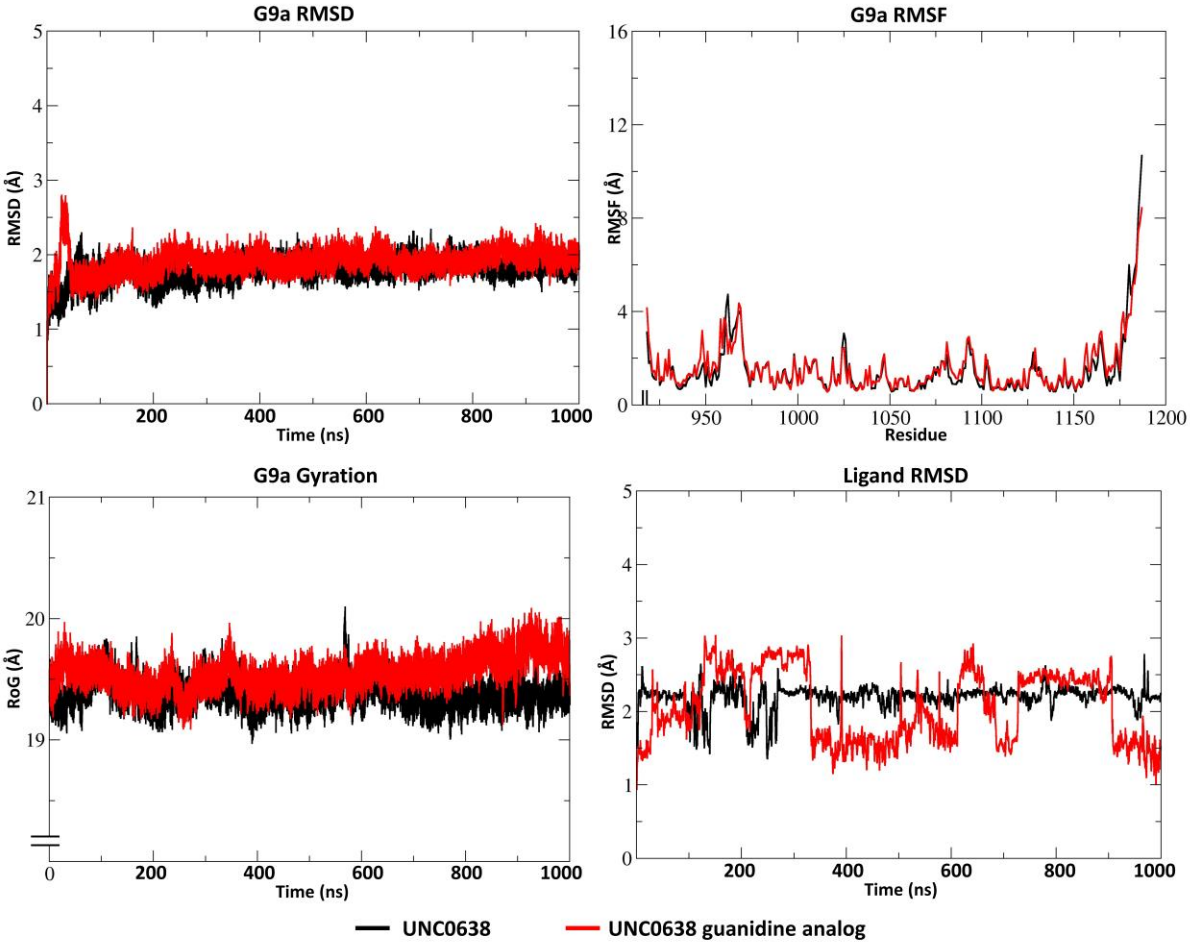

Figure S2. Root mean square deviation (RMSD), root mean square fluctuation (RMSF), radius of gyration and ligand RMSD plots of the UNC0638 and its guanidine analog from 1 μs MD simulation.

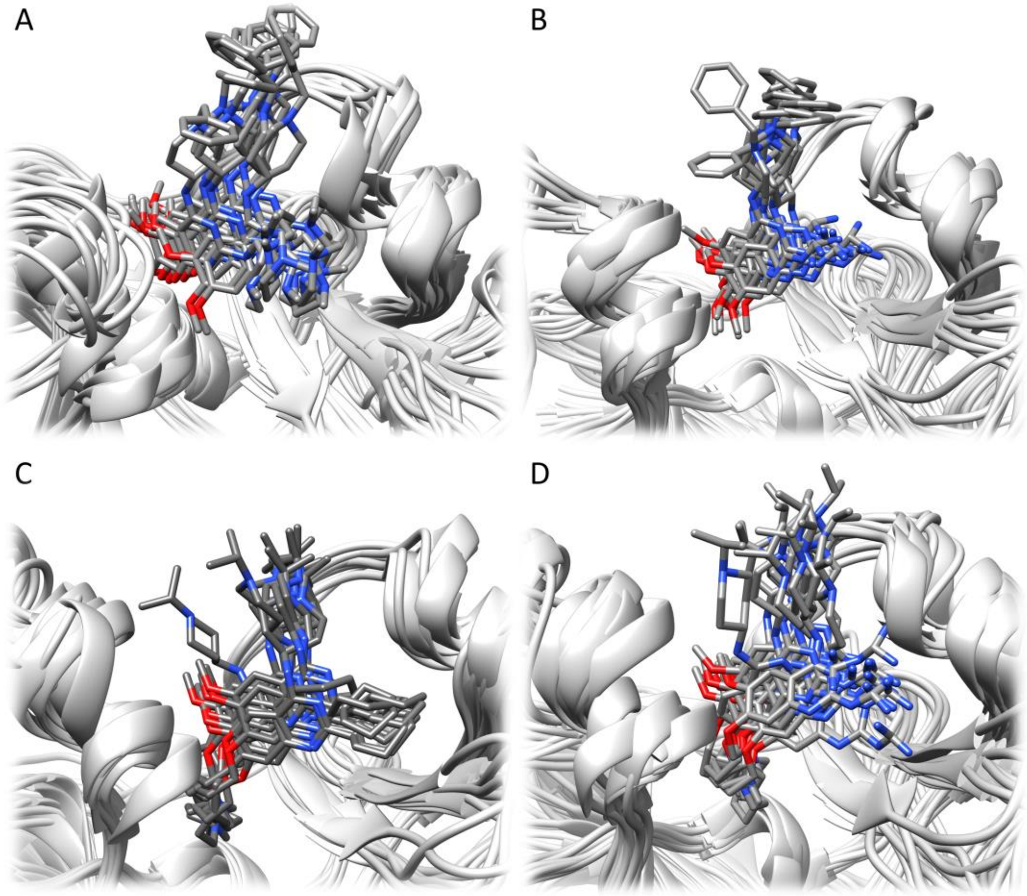

Figure S3. Superimposition of G9a - inhibitor complex structures extracted from molecular dynamics simulations. Frames were extracted at 100 ns intervals from the 1 µs MD trajectory. (A) BIX-01294, (B) BIX-01294 guanidine analog, (C) UNC0638 and (D) UNC0638 guanidine analog.

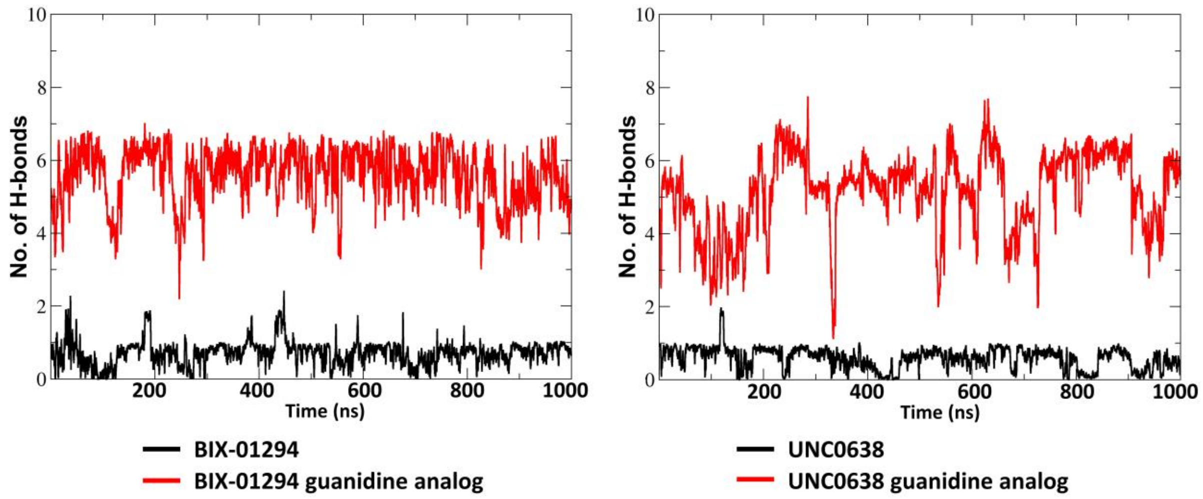

Figure S4. Number of H-bonds (shown as running average plots) observed during molecular dynamics simulation of G9a - inhibitor complexes.

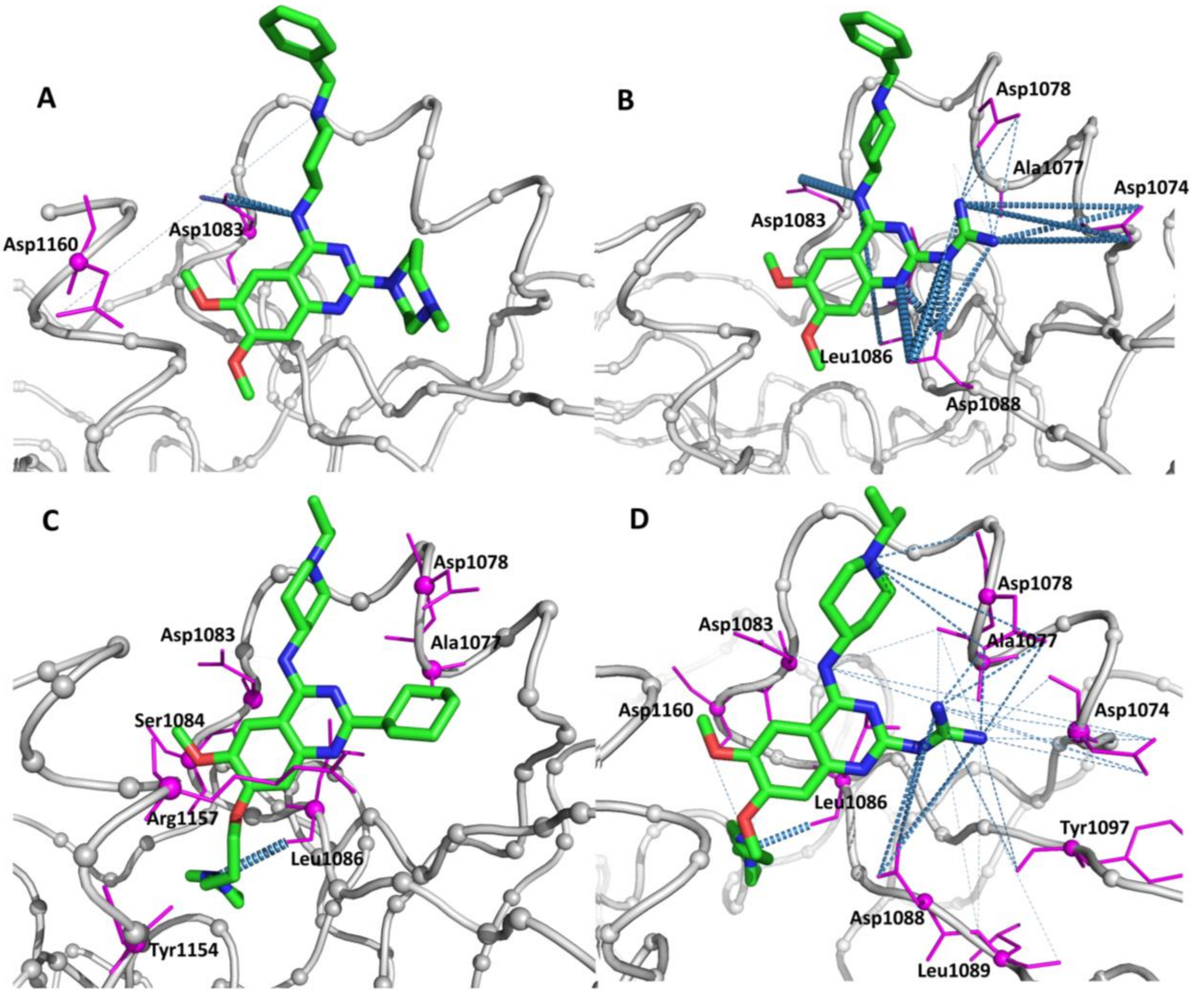

Figure S5. 3D representation of hydrogen bonds (blue dotted line) formed between (A) G9a and BIX-01294, (B) G9a and BIX-01294 guanidine analog, (C) G9a and UNC0638, and (D) G9a and UNC0638 guanidine analog during MD simulation of the G9a - inhibitor complexes. Thickness of the blue dotted lines represents the number of frames during which the particular H-bonds appeared in the MD trajectory. Only H-bonds observed in more than thousand frames of the simulations are shown. H-bond forming residues are shown in magenta solid lines.

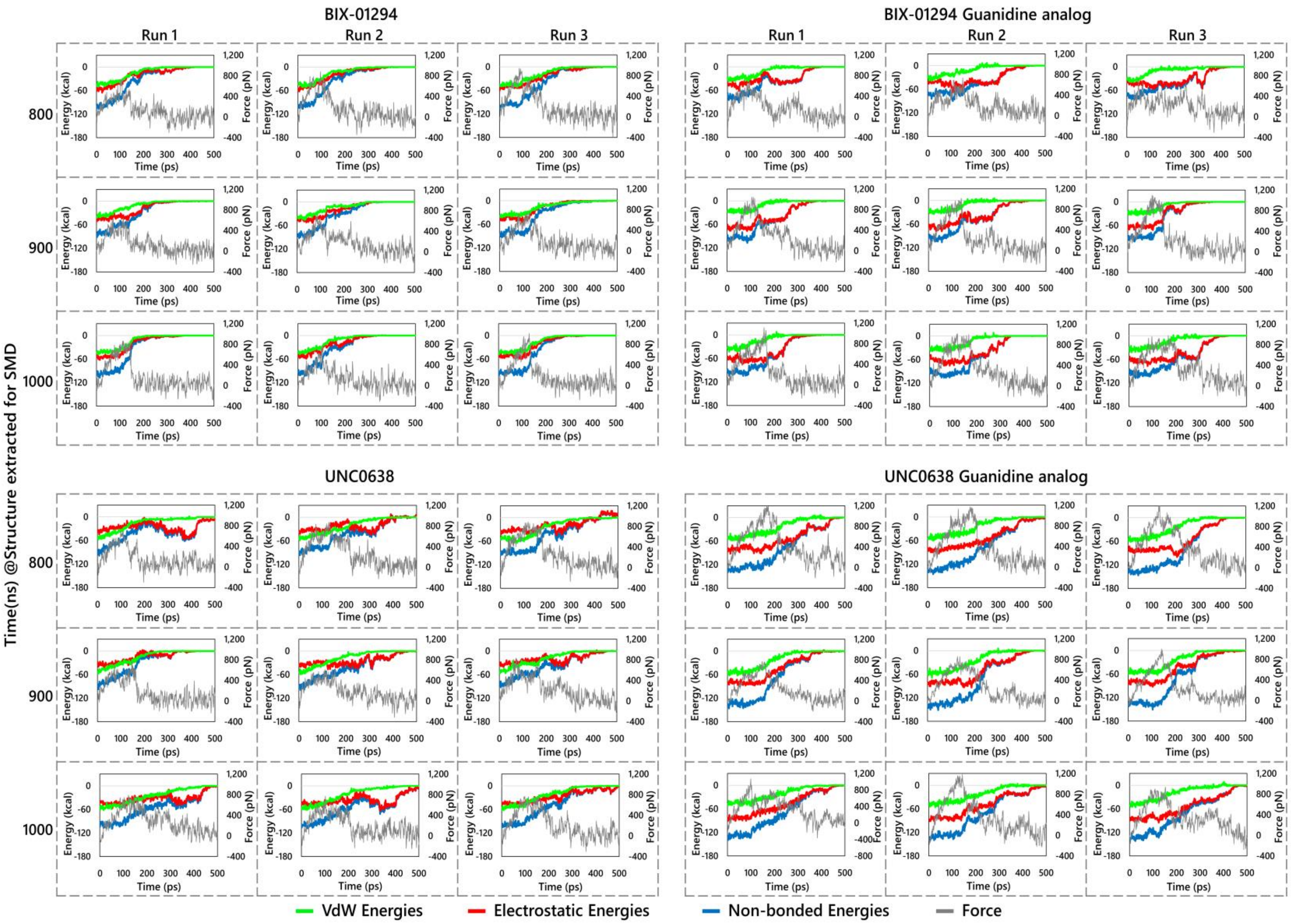

Figure S6. Steered molecular dynamics results of G9a inhibitors and their guanidine analogs.

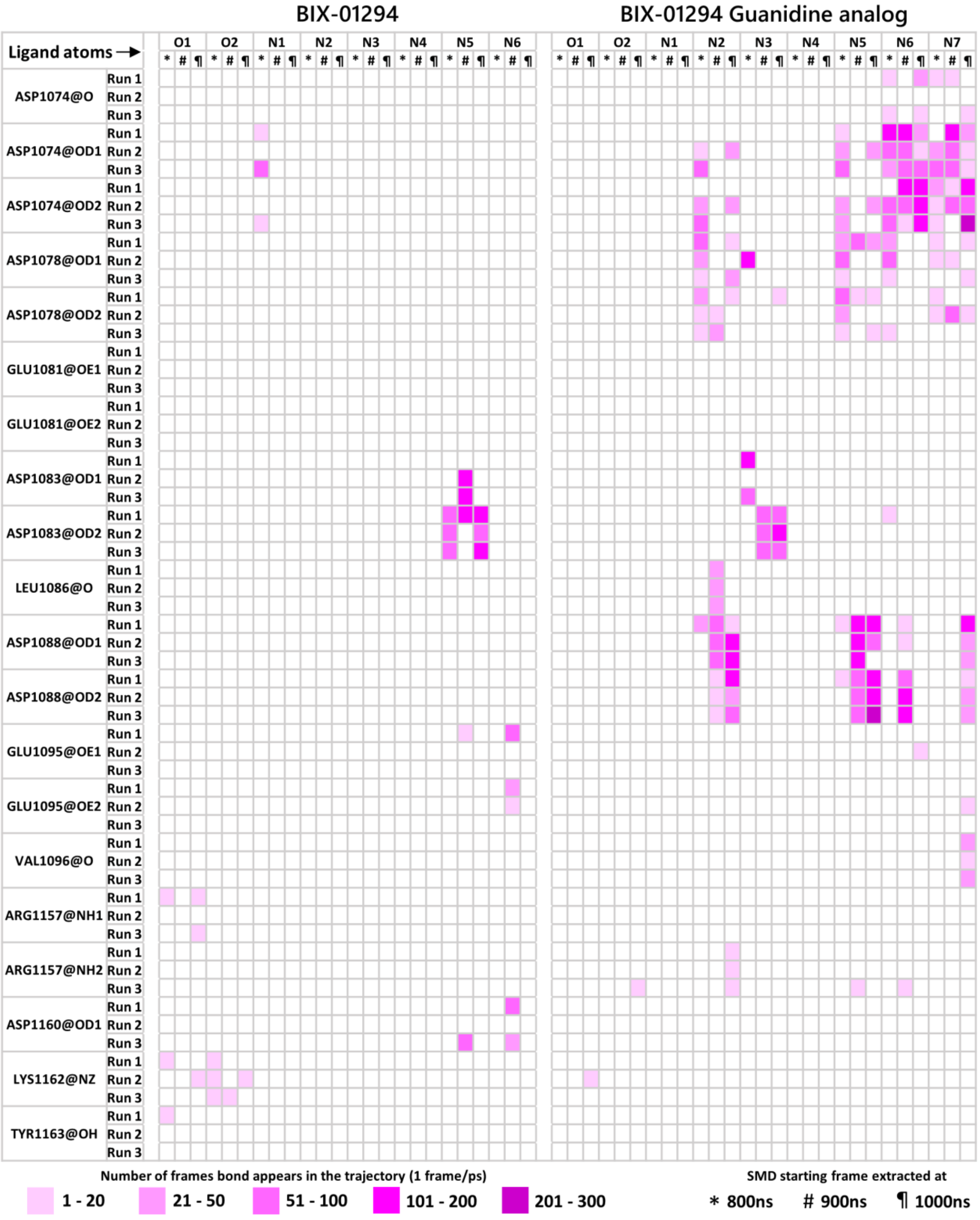

Figure S7. Frequency of H-bonds formed during dissociation of BIX-01294 and BIX-01294 guanidine analog from G9a in SMD experiments.

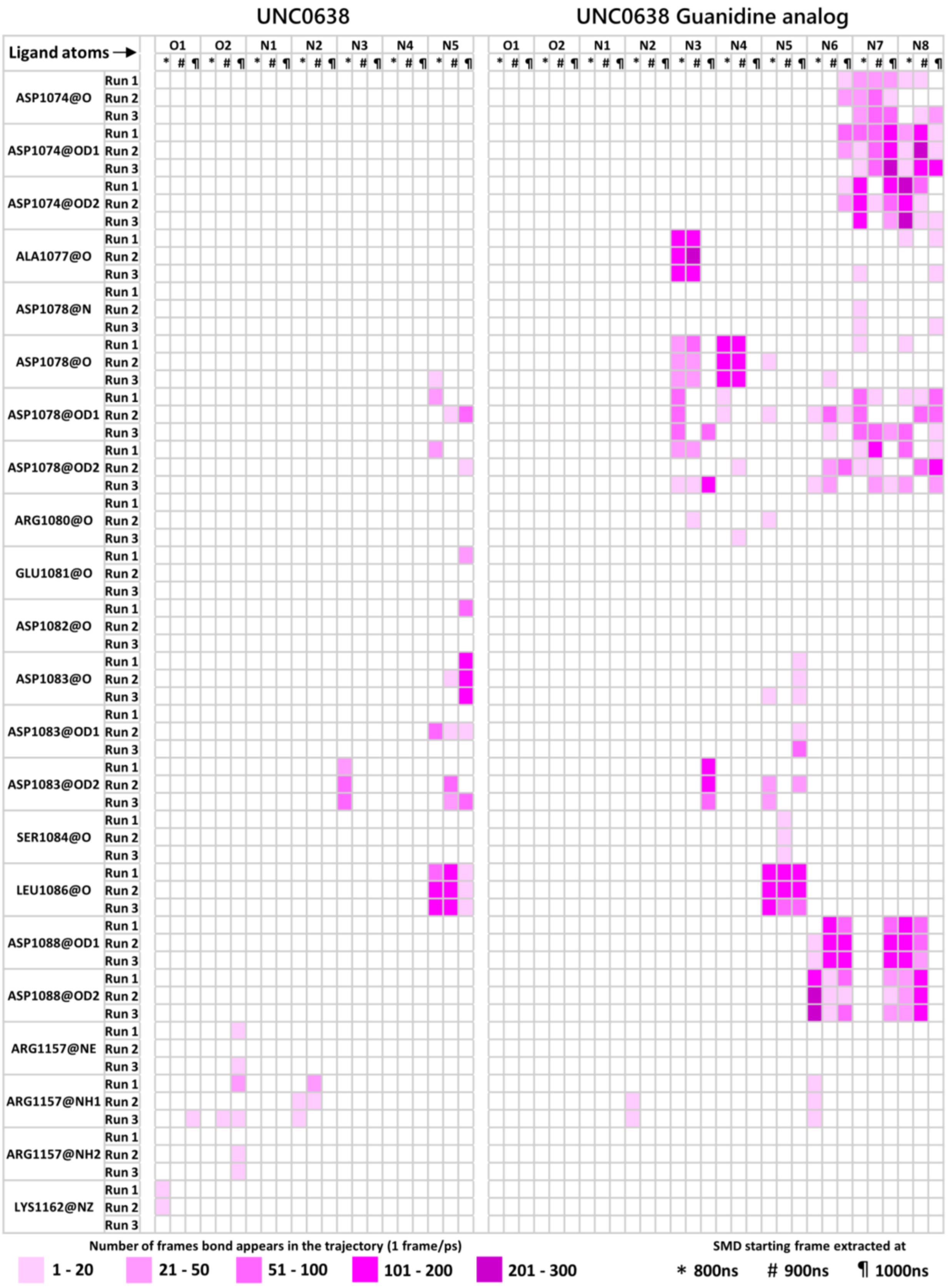

Figure S8. Frequency of H-bonds formed during dissociation of UNC0638 and UNC0638 guanidine analog from G9a in SMD experiments.

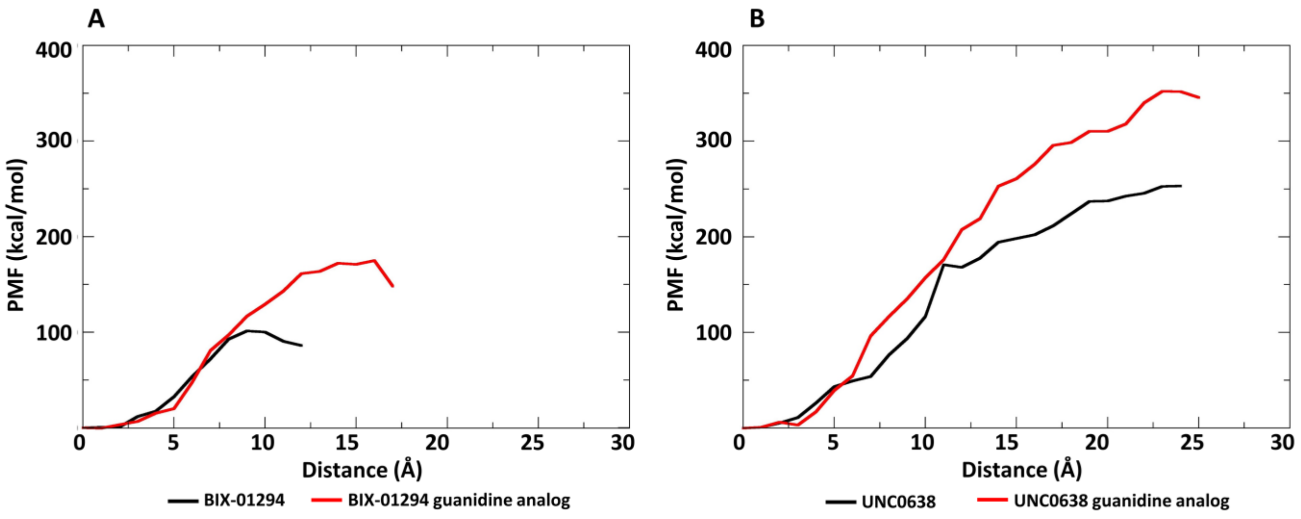

Figure S9. Potential of mean force (PMF) profiles of ligand dissociation for (A) BIX-01294 and BIX-01294 guanidine analog and (B) UNC0638 and UNC0638 guanidine analog.

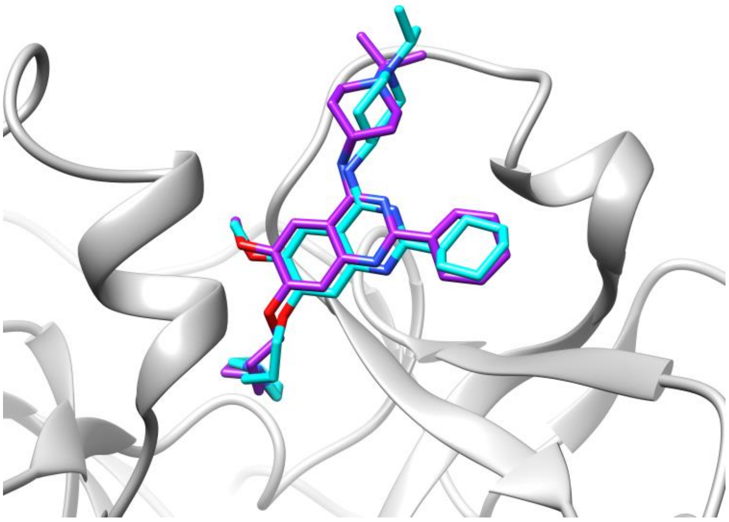

Figure S10. Superimposition of crystal structure (cyan) orientation and docked orientation (magenta) of UNC0638 in G9a protein (PDB ID: 3RJW). RMS deviation of 0.145 Å.
